# A parathyroid hormone/salt-inducible kinase signaling axis controls renal vitamin D activation and organismal calcium homeostasis

**DOI:** 10.1172/JCI163627

**Published:** 2023-05-01

**Authors:** Sung-Hee Yoon, Mark B. Meyer, Carlos Arevalo, Murat Tekguc, Chengcheng Zhang, Jialiang S. Wang, Christian D. Castro Andrade, Katelyn Strauss, Tadatoshi Sato, Nancy A. Benkusky, Seong Min Lee, Rebecca Berdeaux, Marc Foretz, Thomas B. Sundberg, Ramnik J. Xavier, Charles H. Adelmann, Daniel J. Brooks, Anthony Anselmo, Ruslan I. Sadreyev, Ivy A. Rosales, David E. Fisher, Navin Gupta, Ryuji Morizane, Anna Greka, J. Wesley Pike, Michael Mannstadt, Marc N. Wein

**Affiliations:** 1 Endocrine Unit, Massachusetts General Hospital, Harvard Medical School, Boston, Massachusetts, USA.; 2 Department of Nutritional Sciences, University of Wisconsin — Madison, Madison, Wisconsin, USA.; 3 Broad Institute of MIT and Harvard, Cambridge, Massachusetts, USA.; 4 Nephrology Division, Massachusetts General Hospital, Harvard Medical School, Boston, Massachusetts, USA.; 5 Department of Integrative Biology and Pharmacology, McGovern Medical School at The University of Texas Health Science Center at Houston, Houston, Texas, USA.; 6 Université Paris Cité, Institut Cochin, CNRS, INSERM, Paris, France.; 7 Center for Computational and Integrative Biology, Massachusetts General Hospital, Boston, Massachusetts, USA.; 8 Cutaneous Biology Research Center, Department of Dermatology,; 9 Department of Molecular Biology, and; 10 Department of Pathology, Massachusetts General Hospital, Harvard Medical School, Boston, Massachusetts, USA.; 11 Harvard Stem Cell Institute, Cambridge, Massachusetts, USA.; 12 Department of Biochemistry, University of Wisconsin — Madison, Madison, Wisconsin, USA.

**Keywords:** Bone Biology, Nephrology, Calcium, G protein&ndash;coupled receptors, Protein kinases

## Abstract

The renal actions of parathyroid hormone (PTH) promote 1,25-vitamin D generation; however, the signaling mechanisms that control PTH-dependent vitamin D activation remain unknown. Here, we demonstrated that salt-inducible kinases (SIKs) orchestrated renal 1,25-vitamin D production downstream of PTH signaling. PTH inhibited SIK cellular activity by cAMP-dependent PKA phosphorylation. Whole-tissue and single-cell transcriptomics demonstrated that both PTH and pharmacologic SIK inhibitors regulated a vitamin D gene module in the proximal tubule. SIK inhibitors increased 1,25-vitamin D production and renal *Cyp27b1* mRNA expression in mice and in human embryonic stem cell–derived kidney organoids. Global- and kidney-specific *Sik2/Sik3* mutant mice showed *Cyp27b1* upregulation, elevated serum 1,25-vitamin D, and PTH-independent hypercalcemia. The SIK substrate CRTC2 showed PTH and SIK inhibitor–inducible binding to key *Cyp27b1* regulatory enhancers in the kidney, which were also required for SIK inhibitors to increase *Cyp27b1* in vivo. Finally, in a podocyte injury model of chronic kidney disease–mineral bone disorder (CKD-MBD), SIK inhibitor treatment stimulated renal *Cyp27b1* expression and 1,25-vitamin D production. Together, these results demonstrated a PTH/SIK/CRTC signaling axis in the kidney that controls *Cyp27b1* expression and 1,25-vitamin D synthesis. These findings indicate that SIK inhibitors might be helpful for stimulation of 1,25-vitamin D production in CKD-MBD.

## Graphical abstract

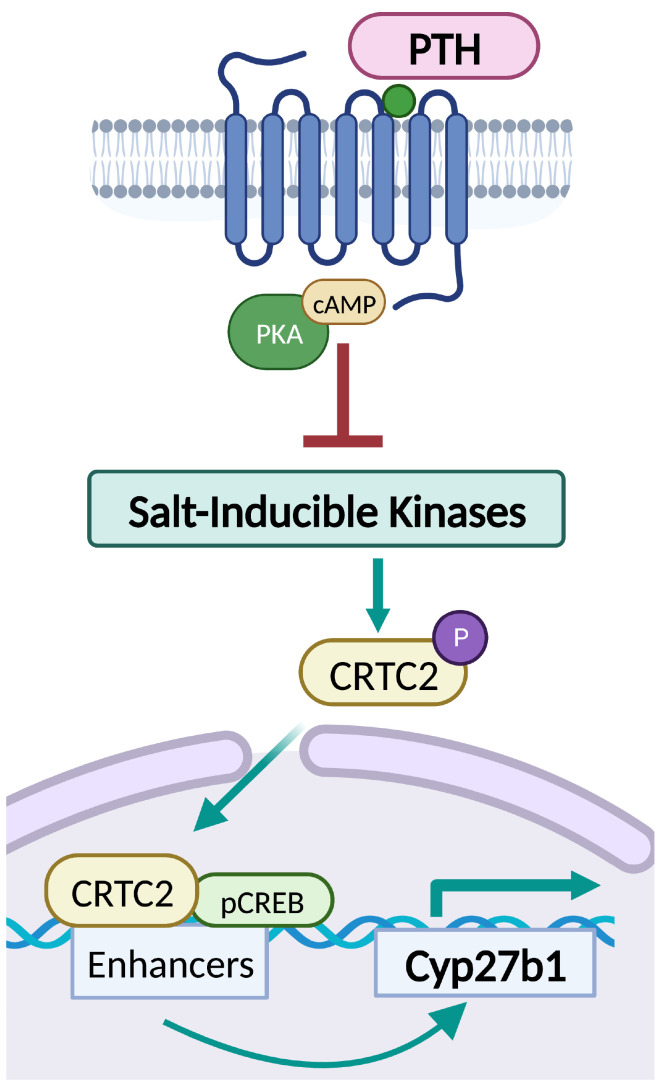

## Introduction

Vitamin D plays a critical role in bone and mineral metabolism and also controls other physiologic and pathophysiologic processes, including skeletal muscle function, reproductive health, cancer risk and progression, diabetes, and autoimmune diseases (1–8). The active metabolite of vitamin D [1,25-(OH)_2_-vitamin D; calcitriol; or 1,25-vitamin D] is mainly produced in the kidney by the cytochrome P450 enzyme 1-hydroxylase (encoded by the *Cyp27b1* gene) under the control of parathyroid hormone (PTH). PTH increases the production of 1,25-vitamin D by increasing *Cyp27b1* and decreasing *Cyp24a1* (the catabolic 24-hydroxylase) expression mainly via an incompletely defined cAMP/PKA-dependent signaling pathway (9–12). Specifically, the steps downstream of PKA activation that drive PTH-dependent *Cyp27b1* gene induction are not known. A kidney-specific enhancer module within the nearby *Mettl1* gene is essential for PTH-induced *Cyp27b1* activation (13). However, how cAMP/PKA signaling regulates this Cyp27b1 enhancer to drive 1,25-vitamin D production is unknown.

Salt-inducible kinases (SIKs) are a subfamily of AMP-activated protein kinases that participate in various cellular processes, including glucose and lipid metabolism, inflammation, melanin production, and bone metabolism (14). Three SIK isoforms have been identified: SIK1, SIK2, and SIK3. SIK1 was first identified in the adrenal gland based on its inducible mRNA expression in response to high-salt diet (15), although its expression has been seen in other tissues, including adipose tissue, brain, muscle, and liver (16–19). In contrast, SIK2 and SIK3 are not regulated by dietary salt intake. Rather, these isoforms are broadly expressed and constitutively activated by their upstream regulator, liver kinase B1 (LKB1) (20). All SIK isoforms are direct substrates of PKA at sites outside their kinase domain. When phosphorylated by PKA, SIK cellular activity is inhibited by an allosteric mechanism that involves 14-3-3 chaperone binding (21–23).

SIKs mediate PTH receptor signaling in osteocytes in bone (24). When PTH binds and activates the PTH receptor, a G protein–coupled receptor, increased cAMP activates PKA, which, in turn, phosphorylates SIKs and suppresses their activity by promoting 14-3-3 protein binding (22). This permits the dephosphorylation of SIK substrates, including class IIa histone deacetylases (HDACs) and CREB-regulated transcriptional coactivator (CRTC) proteins, allowing their translocation into the nucleus to modulate gene expression. SIK gene deletion or pharmacologic inhibition leads to gene expression changes in bone cells, resulting in increased bone formation and dramatic accrual in trabecular bone (24–26).

Besides in bone, the PTH receptor is also abundantly expressed in kidney where its action is crucial to maintain mineral homeostasis by increasing 1,25-vitamin D production, promoting phosphaturia, and stimulating distal renal calcium reabsorption. As SIKs are key factors in the signaling pathways downstream of the PTH receptor in bone, we hypothesized that PTH-induced mineral metabolism changes in the kidneys might be mediated by SIKs. In this study, we demonstrate that pharmacologic SIK inhibitor treatment in vivo and in vitro and global- and kidney-specific SIK gene deletion in mice increased *Cyp27b1* expression and 1,25-vitamin D production and elevated blood calcium levels in vivo. The SIK substrate CRTC2 responded to renal PTH signaling and bound to key *Cyp27b1* enhancers in response to PTH and SIK inhibitor treatment. Furthermore, treatment with a “next-generation” selective SIK2/SIK3 inhibitor stimulated *Cyp27b1* expression and 1,25-(OH)_2_ vitamin D production, even in the setting of chronic kidney disease. Altogether, these findings enrich our current understanding of the signaling pathway downstream of the PTH receptor in the kidney, particularly in terms of *Cyp27b1* regulation, and uncover an important role of SIKs in renal physiology. Furthermore, this work provides insight into therapeutic targeting of SIKs for bone-mineral disorders, including chronic kidney disease–mineral bone disorder (CKD-MBD).

## Results

### SIK inhibitors increase 1,25-vitamin D in mice by increasing renal Cyp27b1 expression.

**Figure 1:**
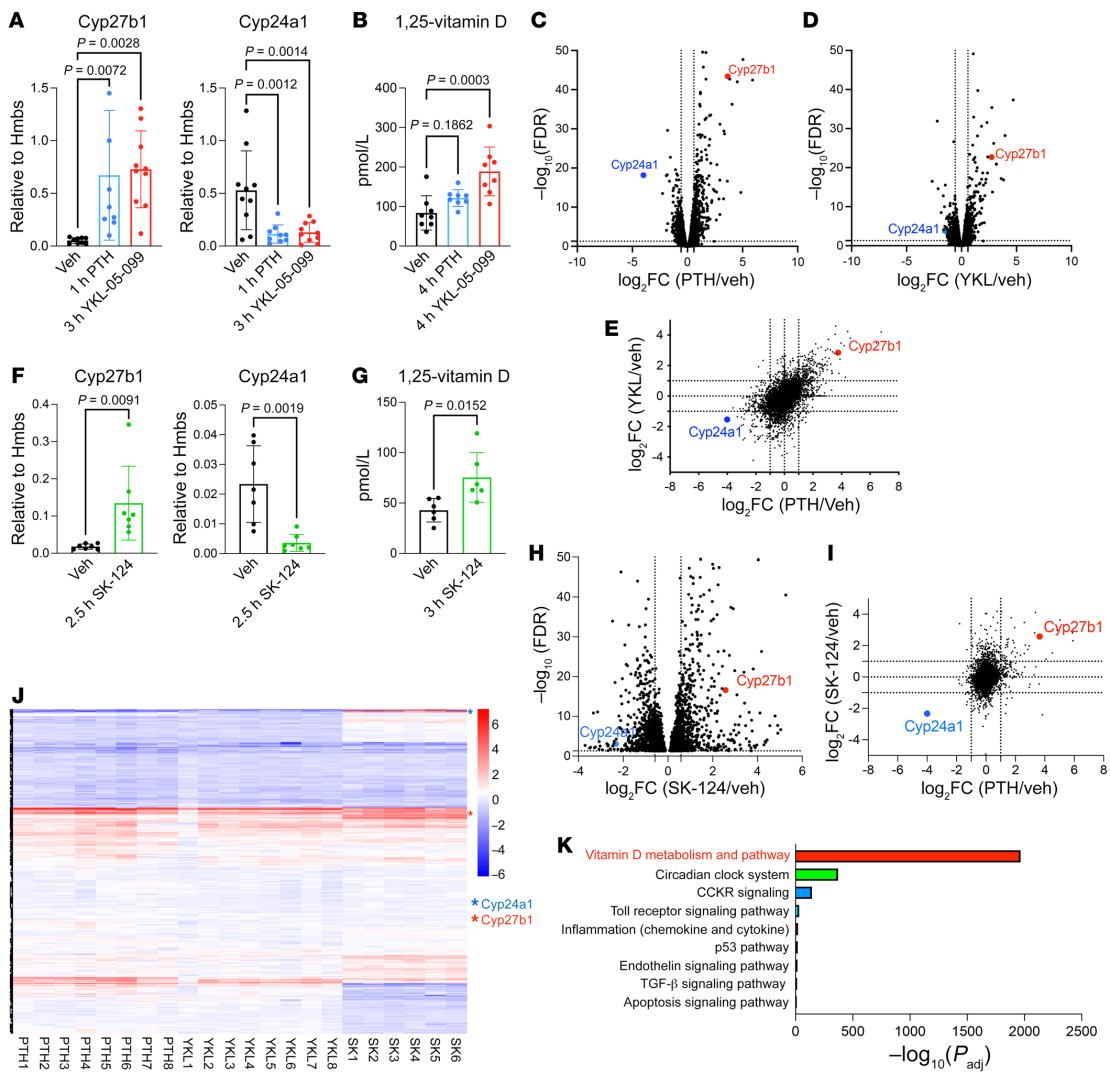
Pharmacologic SIK inhibitors increase renal *Cyp27b1* expression and vitamin D synthesis. (**A** and **B**) 13-week-old male C57BL6 mice were treated with vehicle (Veh), hPTH1-34 (300 μg/kg, s.c.), or YKL-05-099 (45 mg/kg, i.p.), and the animals were sacrificed 1 hour (PTH) or 3 hours (YKL) later for kidney RT-qPCR and 4 hours later for serum 1,25-vitamin D measurements. (**C** and **D**) Whole-kidney bulk RNA-Seq was performed following acute PTH or YKL-05-099 treatment. Volcano plots demonstrating effects of PTH (versus vehicle) and YKL-05-099 (versus vehicle) based upon RNA-Seq show increased *Cyp27b1* and decreased *Cyp24a1* expression, and (**E**) the correlation between these treatments is illustrated in the scatter plot. In an independent study, 8-week-old male C57BL6 mice were treated with vehicle or SK-124 (40 mg/kg, i.p.) and sacrificed after injection at the indicated times for (**F**) gene expression and (**G**) serum 1,25-vitamin D measurement. (**H** and **I**) Bulk RNA-Seq analysis demonstrated increased *Cyp27b1* and reduced *Cyp24a1* expression by SK-124 versus vehicle. (**J**) Heatmap showing normalized expression values (*z* score for each gene versus vehicle) for differentially expressed genes (rows) across individual mice (columns). *Cyp27b1* and *Cyp24a1* are indicated with red and blue asterisks. (**K**) Gene Ontology analysis of up- and downregulated genes by all 3 treatments (PTH, YKL-05-099, and SK-124) showed vitamin D metabolism and pathway as the top GO term. One-way ANOVA with Dunnett’s post hoc test was used for **A** and **B**, and Student’s *t* test was used in **F** and **G**.

First, we sought to determine whether SIKs participate in any of the acute renal actions of PTH. PTH signaling inhibits SIK cellular activity, so direct small-molecule SIK inhibitors serve as valuable tools to study SIK-dependent and -independent aspects of PTH function (24, 26–28). Therefore, 13-week-old C57BL6 mice were treated with vehicle; the “first-generation” pan-SIK inhibitor, YKL-05-099; or PTH, and effects on mineral metabolism were assessed. Like PTH treatment, acute YKL-05-099 administration increased renal *Cyp27b1* and decreased *Cyp24a1* expression and increased circulating 1,25-vitamin D (Figure 1, A and B). Bulk RNA-Seq of these kidneys also showed increased *Cyp27b1* and reduced *Cyp24a1* mRNA levels (Figure 1, C and D). Moreover, differentially expressed genes were similar between YKL-05-099 and PTH treated kidneys, suggesting that a considerable number of PTH-regulated genes are also SIK dependent (Figure 1E; Supplemental Figure 1, A–C; and Supplemental Tables 3 and 4).

Because YKL-05-099 is a relatively promiscuous kinase inhibitor (26), we performed similar studies using SK-124, a selective SIK2/SIK3 inhibitor with improved specificity and bone anabolic action (29). SK-124 also increased *Cyp27b1* and decreased *Cyp24a1* expression and increased circulating 1,25-vitamin D levels (Figure 1, F and G; Supplemental Figure 1G; and Supplemental Table 5). At the transcriptomic level (Figure 1H), SK-124 showed more modest correlation with PTH than YKL-05-099 (Figure 1, I and J, and Supplemental Figure 1, D–F), perhaps because this compound only weakly inhibits SIK1 (29). Gene Ontology analysis of 34 upregulated genes and 1 downregulated gene by all 3 treatment methods (PTH, YKL-05-099, and SK-124) suggested that regulation of vitamin D metabolism is the key action of renal PTH/SIK signaling (Figure 1K), which is the focus of this study. No obvious acute changes in blood or urine calcium and phosphate or circulating PTH levels were noted in initial short-term pharmacologic studies with small-molecule SIK inhibitors (Supplemental Figure 1, H–M). These findings that SIK inhibitors mimic PTH actions in renal vitamin D metabolism suggest that SIKs are important mediators of PTH downstream signaling to stimulate 1,25-vitamin D synthesis.

### Single-cell transcriptomics defines epithelial subsets responsible for PTH and SIK inhibitor Cyp27b1 induction.

**Figure 2:**
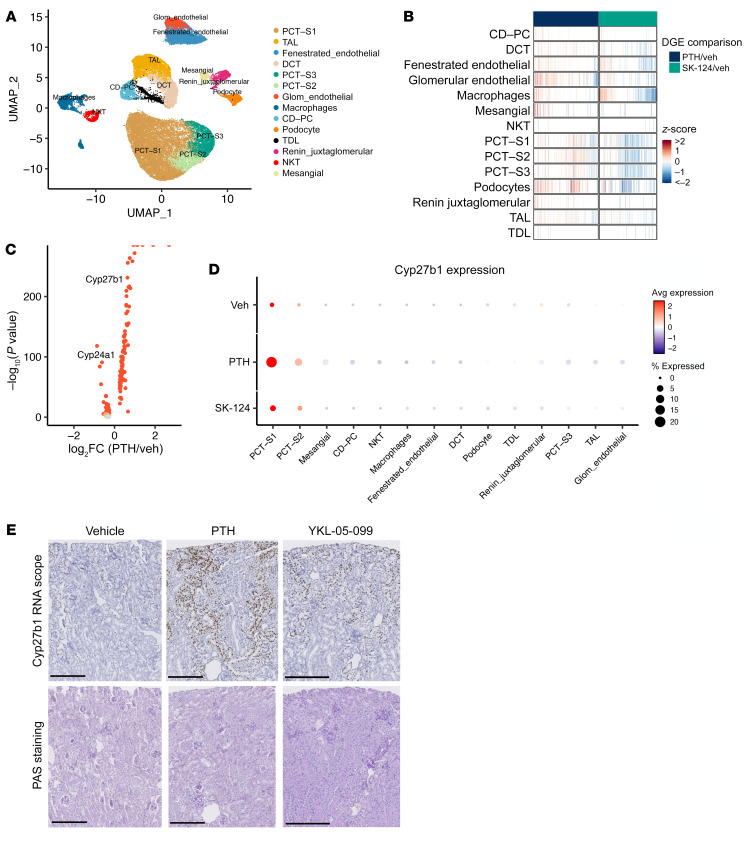
Single-cell RNA-Seq demonstrates cell type–specific renal PTH effects and proximal tubule segment 1–specific *Cyp27b1* induction. (**A**) 8-week-old male C57B6 mice were treated with a single dose of vehicle, PTH (300 μg/kg), or SK-124 (40 mg/kg) and sacrificed 90 minutes or 2.5–3 hours later for PTH or SK-124 treatment, respectively. Single-cell suspensions from kidneys were made for scRNA-Seq. UMAP projection demonstrating expected populations of kidney cells in aggregate data from all 12 samples. (**B**) Heatmap (each line is an individual gene) showing overall patterns of cluster-specific differential gene expression analysis. Distinct groups of genes are regulated by PTH and SIK inhibitors across different renal cell types. (**C**) Volcano plot showing differential gene expression analysis in PCT-S1 cells in response to PTH (versus vehicle). (**D**) Dot plot showing that segment 1 (S1) of proximal convoluted tubule (PCT) is where PTH-induced *Cyp27b1* change is the most prominent. The color of the dots shows average gene expression level, while the size of the dots indicates the percentage of cells expressing the gene of interest in each cluster. (**E**) *Cyp27b1* in situ hybridization (brown, RNAscope) in paraffin-embedded kidney sections. Periodic acid–Schiff stain was performed to mark tubular morphology. Scale bars: 250 μm.

Previous studies have used immunohistochemistry and in situ hybridization to attempt to localize the cell types in kidney in which PTH induces *Cyp27b1* expression. Moreover, single-cell chromatin accessibility profiling has suggested proximal tubule–specific regulatory regions that may control hormonal *Cyp27b1* regulation (30). Together, these findings have suggested that PTH induces 1,25-vitamin D synthesis in the proximal tubule, though precise cellular resolution of this key endocrine action of PTH has remained elusive (31). To define the cell population responsible for PTH- and SIK-regulated *Cyp27b1* induction, we performed droplet-based single-cell RNA-Seq (scRNA-Seq) in kidneys of mice treated acutely with vehicle, PTH, or SK-124 (*n* = 4 per treatment group). After applying quality control cutoffs, a total of 42,820 cells were analyzed (*n* = 15,209 cells from vehicle-treated mice; 14,219 from PTH-treated mice; and 13,392 from SK-124–treated mice). Supervised clustering using well-accepted kidney cell markers (32) demonstrated expected populations of epithelial, stromal, endothelial, and immune cell populations (Figure 2A and Supplemental Figure 2A). As expected, overall cluster cellularity was not affected by acute PTH or SK-124 treatment (Supplemental Figure 2, B and C). In contrast, cluster-specific differential gene expression analysis revealed multiple groups of transcripts within distinct renal cell populations with expression that was acutely regulated by PTH or SK-124 (Figure 2B and Supplemental Tables 6 and 7). Consistent with broad gene regulatory effects of PTH throughout the kidney, PTH receptor (*Pth1r*) mRNA was highly expressed in both proximal and distal epithelial cells and in podocytes (Supplemental Figure 2D). *Cyp27b1* induction and *Cyp24a1* suppression by PTH occurred predominantly in proximal convoluted tubule S1 subsegment cells (PCT-S1) (Figure 2C and Supplemental Figure 2, E and F). The SIK2/SIK3 inhibitor SK-124 also showed PTH-like effects, including *Cyp27b1* upregulation and Cyp24a1 downregulation in S1 segment of proximal tubules, although the degree of these changes was more modest than those seen after PTH treatment (Figure 2D and Supplemental Figure 2, E–G). In situ hybridization validated these scRNA-Seq data and demonstrated prominent *Cyp27b1* induction in glomeruli-adjacent proximal tubular cells (Figure 2E). Taken together, these findings demonstrate that PTH stimulates broad cell type–specific patterns of gene expression changes in renal Pth1r-expressing cells and that PTH-induced *Cyp27b1*/*Cyp24a1* regulation occurs predominantly in PCT-S1.

### PTH/SIK signaling in human embryonic stem cell–derived kidney organoids increases Cyp27b1 expression and 1,25-vitamin D synthesis.

**Figure 3:**
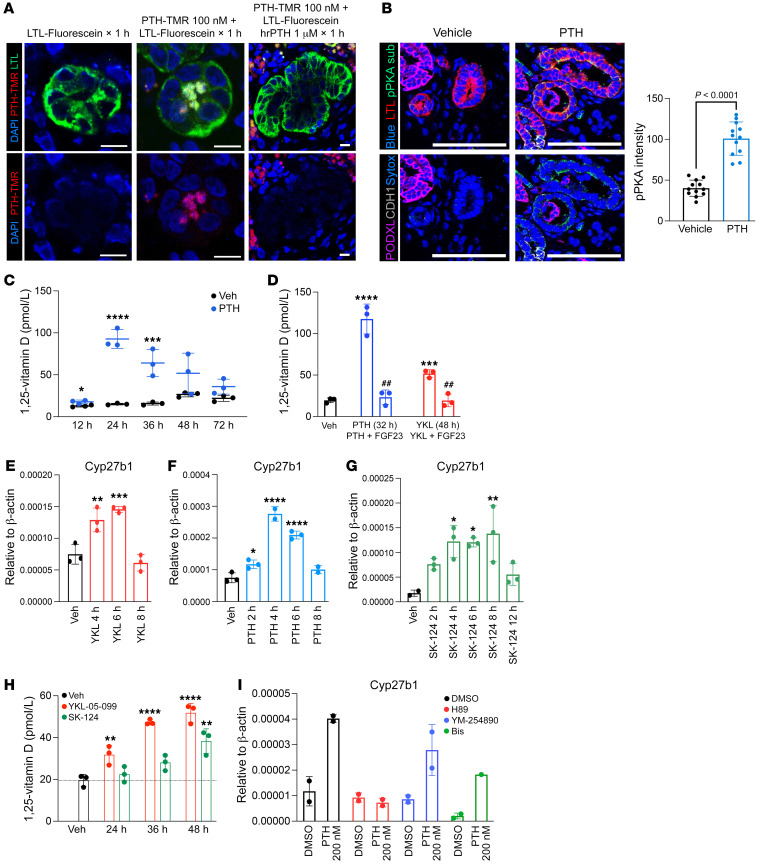
PTH and SIK inhibitors increase *Cyp27b1* expression and 1,25-vitamin D synthesis in human kidney organoids. (**A**) H9 human embryonic stem cell–derived kidney organoids were treated with TMR-labeled PTH and then stained with LTL. (**B**) Kidney organoids were treated with PTH1-34 for 30 minutes and immunostained with pPKA substrate antibody to show PTH responsiveness (Lotus Tetragonolobus Lectin [LTL] for proximal tubule; CDH1 for distal tubule; podocalyxin [PODXL] for podocytes; Sytox Blue as the nuclear dye). Scale bars: 100 μm. Quantification is shown in the histogram on the right. Student’s *t* test was performed for statistical analysis. (**C**) Day 28 organoids were treated for the indicated times with vehicle or PTH (242 nM), and 1,25-vitamin D was measured in the culture media by ELISA. (**D**) PTH-induced 1,25-vitamin D production was inhibited by cotreatment with FGF23 (100 nM). (**E**–**G**) *Cyp27b1* gene expression was measured by RT-qPCR in response to treatment for the indicated times with PTH (242 nM) and SIK inhibitors, YKL-05-099 (10 μM) and SK-124 (20 μM). (**H**) Similar experiments in which 1,25-vitamin D was measured in the culture media by ELISA. (**I**) Pretreatment with H89 (10 μM), YM-254890 (10 μM), and bisindolylmaleimide I (10 μM) for 1 hour prior to 4-hour PTH treatment (242 nM) showed that cAMP/PKA pathway is responsible for PTH-induced *Cyp27b1* upregulation. Data are shown as the mean ± SD, and each dot represents independent sample of 3–4 organoids pooled together. For **C**, Student’s *t* test were performed between PTH and Veh at each time point. For **D**, Student’s *t* test was performed against Veh (indicated by ***P < 0.001, ****P < 0.0001) and to test the effect of FGF23 (indicated by ^##^*P* < 0.001). For **E**–**H**, 1-way ANOVA was performed, with Dunnett’s post hoc test, against Veh (*P < 0.05; **P < 0.01, ***P < 0.001, ****P < 0.0001).

Because no kidney proximal tubule cell line that shows PTH-regulated *Cyp27b1* expression and 1,25-vitamin D synthesis has been reported, kidney organoids generated from H9 human embryonic stem cells were used (33). In this system, organoid maturation is accompanied by progressive upregulation of genes involved in mineral metabolism, including *Pth1r*, *Cyp27b1*, and *Slc34a1* (Supplemental Figure 3, A–C). We confirmed functional PTH receptor expression by specific binding of tetramethylrhodamine-labeled (TMR-labeled) PTH (1-34) in Lotus Tetragonolobus Lectin–labeled (LTL-labeled) proximal tubule cells (Figure 3A) in a pattern consistent with in vivo TMR-PTH staining (34). In addition, organoid PTH treatment led to accumulation of phosphorylated PKA substrates in proximal tubule cells (Figure 3B), *Cyp27b1* mRNA upregulation (Figure 3E), and accumulation of 1,25-vitamin D in culture media (Figure 3C). Importantly, we noted opposing hormonal regulation of 1,25-vitamin D synthesis by FGF23 (Figure 3D). Similar to their actions in vivo, the SIK inhibitors YKL-05-099 and SK-124 also triggered *Cyp27b1* expression and increased 1,25-vitamin D (Figure 3, E–H). These data support the utility of human kidney organoids as a tool to study the regulation of vitamin D metabolism by hormonal cues.

PTH receptor stimulation can lead to increases in several second messengers, including cAMP/PKA and PKC/IP_3_-iCa^2^ (35). To test which signaling arm of PTH receptor is responsible for increasing *Cyp27b1* expression, organoids were treated with DMSO, H89 (PKA inhibitor), YM-254890 (Gαq/11 inhibitor), or bisindolylmaleimide (PKC inhibitor) prior to PTH treatment. In agreement with current literature (36–38), H89 pretreatment blocked PTH-induced *Cyp27b1* expression (Figure 3I), confirming that a SIK-dependent increase in *Cyp27b1* expression by PTH in human kidney organoids is predominantly regulated through the Gs/cAMP/PKA signaling arm, rather than the PKC pathway.

### Global, inducible SIK mutant mice show increased 1,25-vitamin D and Cyp27b1 expression, with PTH suppression.

**Figure 4:**
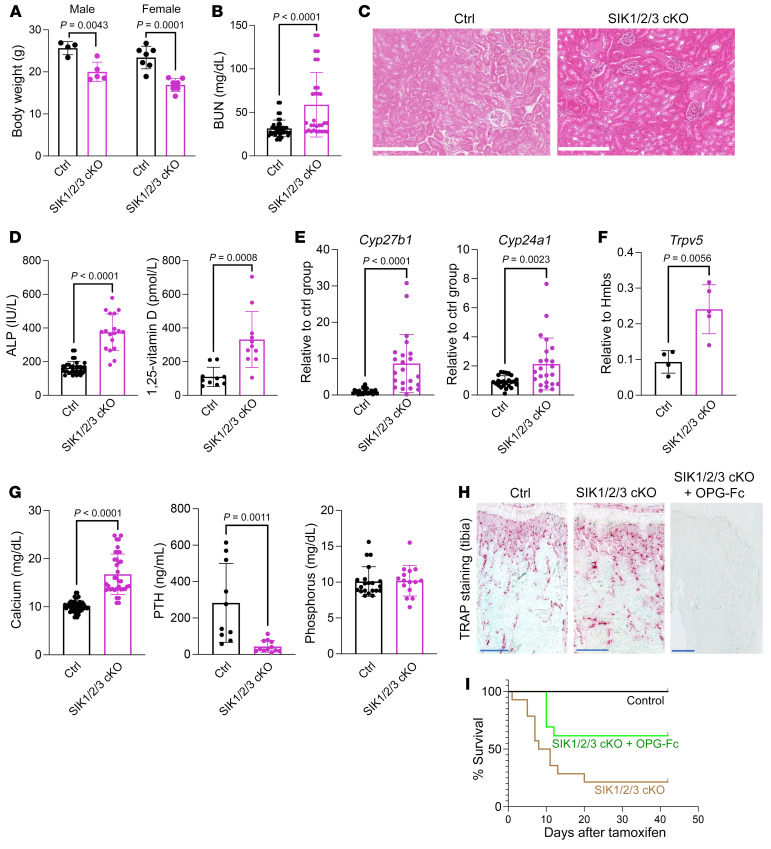
Global/inducible deletion of all 3 SIK isoforms causes PTH-independent hypercalcemia and lethality. (**A**) 6-week-old mice of the indicated genotypes were treated with tamoxifen. Body weights of male and female inducible SIK triple-KO (iTKO) mice were obtained at the time of sacrifice. (**B**) Blood urea nitrogen (BUN) measurement in control and iTKO mice. (**C**) Renal histology (H&E stain) from control and iTKO animals revealing no obvious differences between genotypes. Scale bars: 250 μm. (**D**) Alkaline phosphatase (ALP) and 1,25-vitamin D measurement in control and iTKO mice. (**E** and **F**) Renal gene expression of *Cyp27b1*, *Cyp24a1*, and *Trpv5* by RT-qPCR. (**G**) Serum analysis of calcium, PTH, and phosphorus in control and iTKO mice. (**H**) TRAP staining of decalcified, paraffin-embedded tibia sections reveals increased osteoclasts in iTKO mice. Osteoclasts are essentially absent following OPG-Fc treatment. Scale bars: 500 μm. (**I**) Survival curve of iTKO mice treated with or without OPG-Fc (200 μg/mouse) (*n* = 42 Ctrl, *n* = 14 iTKO, *n* = 13 iTKO + OPG-Fc). Data are shown as the mean ± SD, and each dot represents an individual mouse. Student’s *t* test was performed for statistical analysis, and *P* values are shown.

To complement our pharmacologic studies with SIK inhibitors, we created global, tamoxifen-inducible SIK mutant mice. Ubiquitin-Cre^ERt2^;*Sik1*^fl/fl^;*Sik2*^fl/fl^;*Sik3*^fl/fl^ mice lost body weight shortly after tamoxifen injection (Figure 4A). These mice had elevated blood urea nitrogen (BUN) levels without obvious renal histopathology (Figure 4, B and C) and poor survival rate (see below). Analysis of surviving mice revealed expected changes in bone remodeling (25) (Figure 4, D and H). Inducible SIK triple-KO (iTKO) mice also showed increased serum 1,25-vitamin D levels due to increased renal *Cyp27b1* expression (Figure 4, D and E). In contrast to effects of short-term pharmacological SIK inhibition, *Cyp24a1* expression was increased in SIK-KO mice (Figure 4E), likely due to chronically elevated 1,25-vitamin D, which is a potent inducer of both *Cyp24a1* (39–41) and *Trpv5* (Figure 4F) (42). iTKO mice showed dramatic hypercalcemia (Figure 4G) with suppressed PTH levels (Figure 4G), likely due to high 1,25-vitamin D levels and increased bone resorption. Interestingly, serum phosphorus levels were comparable between control and iTKO mice (Figure 4G). We hypothesized that hypercalcemia was responsible for the poor survival rate in these mice. Therefore, we treated SIK iTKO mice with OPG-Fc, a potent inhibitor of bone resorption and, therefore, skeletal calcium release (Figure 4, H and I). Indeed, OPG-Fc partially rescued accelerated lethality in iTKO animals (Figure 4I), suggesting that hypercalcemia contributes in part to the accelerated mortality seen upon global/inducible deletion of all 3 SIK isoforms.

### “Pseudohyperparathyroidism” in kidney-specific SIK mutant mice.

**Figure 5:**
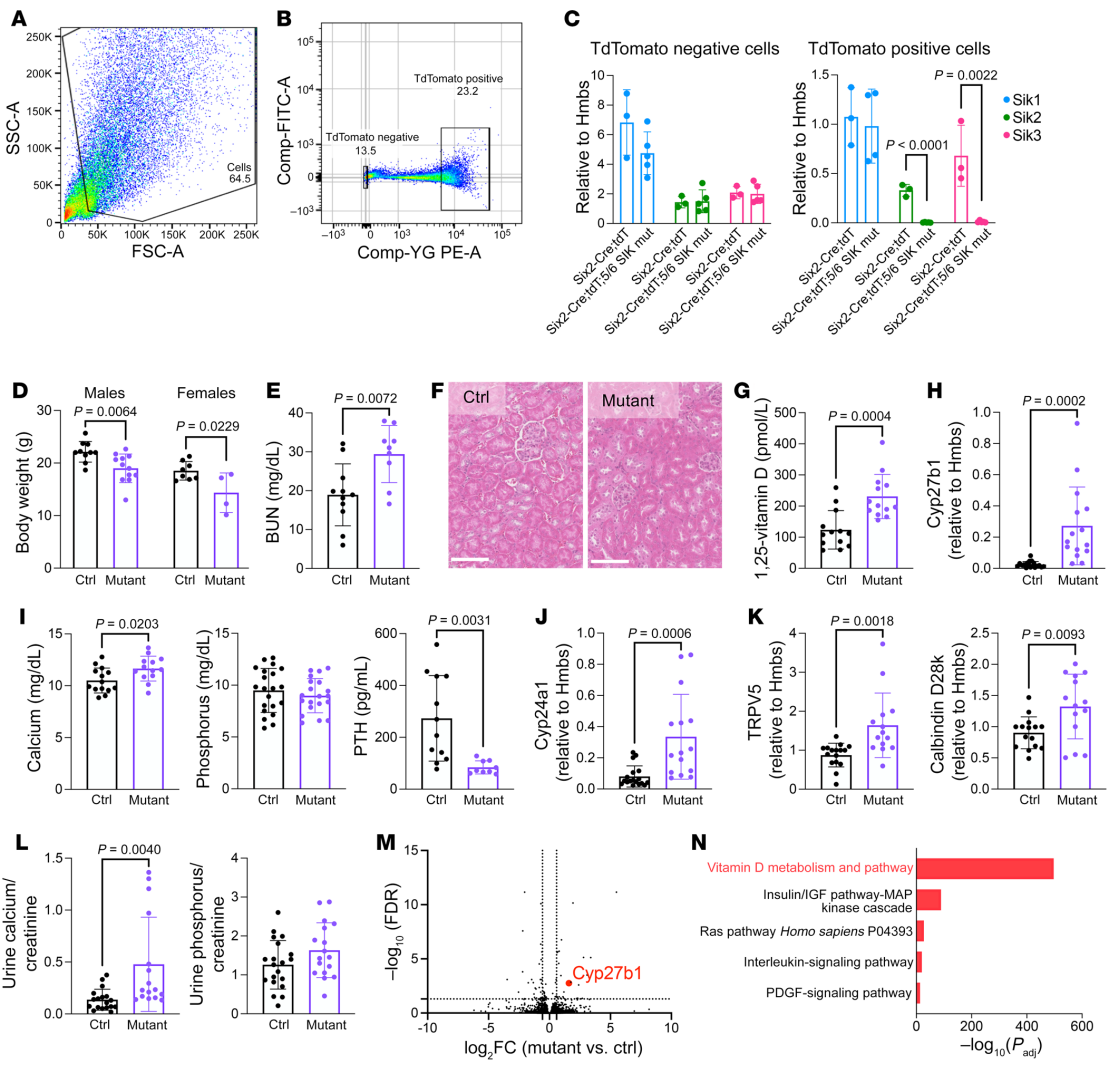
Renal pseudohyperparathyroidism upon kidney-specific SIK deletion. (**A** and **B**) Six2-Cre and Six2-Cre;*Sik1*^fl/+^;*Sik2*^fl/fl^;*Sik3*^fl/fl^ mice were crossed with Ai14 tdTomato reporter mice, and tdTomato-positive cells were sorted to assess gene deletion efficiency more accurately without other cell types, including blood and immune cells. Single-cell suspensions from kidneys were generated, and tdTomato-positive and -negative cells were sorted as shown. (**C**) tdTomato-positive cells showed approximately 98% *Sik2* and *Sik3* gene deletion efficiency in Six2-Cre;*Sik1*^fl/+^;*Sik2*^fl/fl^;*Sik3*^fl/fl^ mice, and tdTomato-negative cells did not show any change in *Sik* mRNA expression levels. (**D**) Body weights and (**E**) serum blood urea nitrogen (BUN) are shown, (**F**) along with kidney histology (H&E stain) images from the indicated animals. No overt histologic differences were noted in mutants. Scale bars: 100 μm. (**G**) 1,25-vitamin D measurement in control and SIK mutant mice. (**H**) Renal Cyp27b1 expression by RT-qPCR. (**I**) Serum calcium, phosphorus, and PTH measurements are shown. (**J** and **K**) Renal RNA was isolated to measure 1,25-vitamin D–induced genes (*Cyp24a1*, *TRPV5*, and *Calbindin D28k* [*Calb1*]) by RT-qPCR. (**L**) Urine calcium and phosphorus are normalized to creatinine. (**M**) Bulk RNA-Seq from whole-kidney RNA from control and mutant mice showed increased *Cyp27b1*. (**N**) GO terms from dysregulated genes in SIK mutant mice. Data are shown as the mean ± SD, and each dot represents an individual mouse. Student’s *t* test was performed for statistical analysis, and *P* values are shown.

Skeletal SIK deletion in global iTKO mice likely contributed to hypercalcemia and early lethality. Therefore, we generated kidney-specific SIK mutant mice by crossing mice with a series of loxP-flanked (floxed) SIK isoforms with Six2-Cre mice (43) (Supplemental Figure 4). We were unable to generate surviving Six2-Cre;*Sik1*^fl/fl^;*Sik2*^fl/fl^;*Sik3*^fl/fl^ triple-KO mice. Instead, we first analyzed Six2-Cre;*Sik1*^fl/+^;*Sik2*^fl/fl^;*Sik3*^fl/fl^ mice to evaluate the deletion of 5 of 6 SIK alleles in the kidney. Whole-kidney (including hematopoietic and stromal cells that lack Six2-Cre activity) DNA and mRNA analysis in these animals showed approximately 50% deletion efficiency (Supplemental Figure 5, A and B). However, in sorted tdTomato^+^ cells, which showed increased *Cyp27b1* mRNA (Supplemental Figure 5C), *Sik2* and *Sik3* gene deletion efficacy was more than 98%, as assessed by RT-qPCR (Figure 5, A–C). Mutant mice were born smaller compared with their littermate controls, a phenotype that became less dramatic with age (Figure 5D and Supplemental Figure 6). BUN levels were statistically higher in SIK mutants than controls but still were within the normal mouse reference range (Figure 5E). We detected no obvious renal histological abnormalities (Figure 5F). At present we cannot distinguish between BUN overproduction and slight reduction in eGFR due to hypercalcemia as the cause of elevated BUN in these animals. Similar to pharmacologic studies with systemic SIK inhibitors, renal SIK deletion also led to increased serum 1,25-vitamin D (Figure 5G) due to elevated renal *Cyp27b1* expression (Figure 5H). Ultimately, these changes led to mild PTH-independent hypercalcemia (Figure 5I), a renal pseudohyperparathyroidism phenotype likely caused by downstream activation of the PTH receptor signaling cascade by SIK gene deletion. Further evidence supporting biologic actions of high 1,25-vitamin D included changes in renal *Cyp24a1*, *Trpv5*, and *Calbindin D28k* expression (Figure 5, J and K); circulating intact and C-terminal FGF23 levels (Supplemental Figure 6, W and X); and hypercalciuria (Figure 5L). Despite increased renal *Cyp24a1* mRNA and circulating FGF23, which both inhibit the production of 1,25-vitamin D (44, 45), serum 1,25-vitamin D levels were still elevated, demonstrating a strong stimulatory effect of renal SIK deletion on vitamin D metabolism despite counterregulatory mechanisms.

To complement ELISA-based 1,25-vitamin D measurements, we also confirmed that 1,25-vitamin D, but not other vitamin D metabolites, was high in SIK mutants (Supplemental Figure 5D) using LC-MS/MS. Age and sex did not influence the mineral metabolism phenotype (Supplemental Figure 6). Importantly, SK-124 treatment did not further increase *Cyp27b1* expression in Six2-Cre;*Sik1*^fl/+^;*Sik2*^fl/fl^;*Sik3*^fl/fl^ mice, suggesting that this agent stimulates *Cyp27b1* induction via targeting renal SIK isoforms (Supplemental Figure 7). Renal PTH actions promote phosphaturia (46). However, we did not observe changes in blood or urine phosphate levels upon SIK deletion in global/inducible (Figure 4G) and renal-specific (Figure 5, I and L and Supplemental Figure 6, U and Z) SIK mutant models. Moreover, PTH, but not SK-124, reduced Npt2a immunostaining in human kidney organoids and blocked Npt2a-dependent phosphate uptake in cultured opossum kidney cells (Supplemental Figure 8, A and B). Taken together, these observations suggest that SIKs may not be involved in the renal phosphaturic actions of PTH.

We assessed the overall transcriptomic effects of renal SIK gene deletion by performing bulk RNA-Seq (Supplemental Table 8). *Cyp27b1* was 1 of 27 upregulated genes (Figure 5M), and “vitamin D metabolism” was the top GO term when 57 dysregulated genes were analyzed (FDR < 0.05; FC > 1.5) (Figure 5N). Thus, kidney-specific SIK deletion in mice resulted in *Cyp27b1* stimulation, which led to PTH-independent, 1,25-vitamin D–dependent hypercalcemia. These results provide important genetic confirmation that SIKs are involved in renal 1,25-vitamin D synthesis.

### Osteopenia and increased bone resorption in renal-specific SIK mutant mice.

Bone-specific SIK deletion leads to increased bone formation and trabecular bone mass (25). These results indicate that the skeletal actions of PTH preferentially stimulate bone formation. Generation of renal-specific SIK mutant mice provides an opportunity to test the effects of renal pseudohyperparathyroidism on bone. As predicted from elevated 1,25-vitamin D levels, Six2-Cre*;Sik1*^fl/+^;*Sik2*^fl/fl^;*Sik3*^fl/fl^ mice showed increased serum bone turnover markers (P1NP and CTx) compared with control littermates (Supplemental Figure 9, A and B) and mildly reduced cortical bone mineral density (Supplemental Figure 9, C–I). Because osteoclast numbers were unchanged yet osteoclast surfaces were increased (Supplemental Figure 9, J–L), it is likely that high circulating 1,25-vitamin D levels drive osteoclastogenesis by increasing RANKL, decreasing OPG, and increasing the expression of osteoclastic adhesion molecule, αVβ3 integrin (47). Larger osteoclasts are known to possess more resorbing activity than smaller osteoclasts (48–50). Six2-Cre*;Sik1*^fl/+^;*Sik2*^fl/fl^;*Sik3*^fl/fl^ bones were shorter than those of control littermates, as expected from the smaller body size of these mutants (Supplemental Figure 9H and Figure 5, G and H). Overall, this analysis confirms endocrine actions of high 1,25-vitamin D in renal-specific SIK mutant mice on bone and suggests that the renal actions of PTH may mitigate the ability of this hormone, over the long term, to optimally boost bone mass.

### Identification of SIK2 and SIK3 as the isoforms responsible for Cyp27b1 regulation.

**Figure 6:**
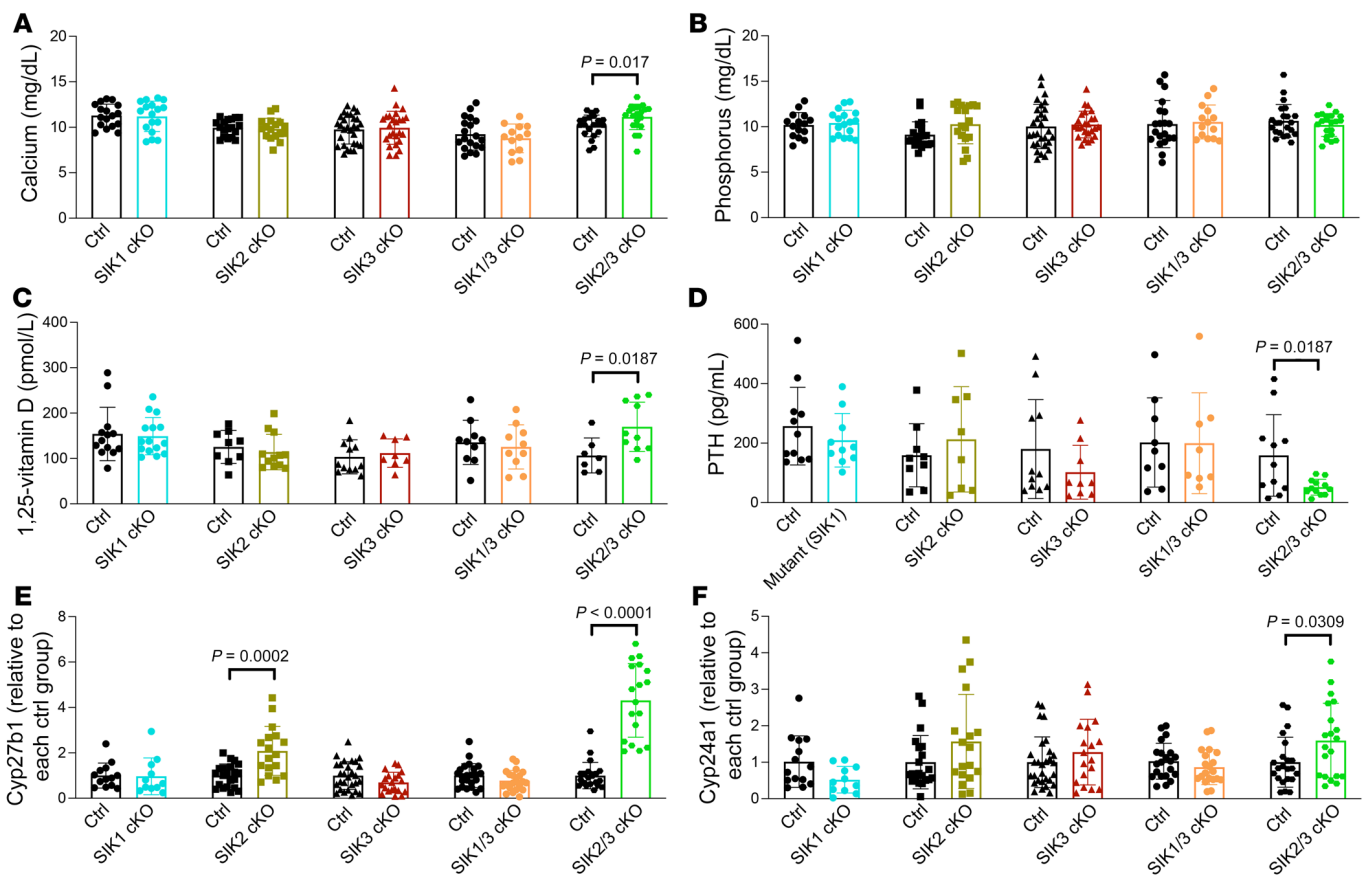
Allelic series of kidney-specific SIK mutant mice reveals a key role for SIK2 and SIK3 in controlling *Cyp27b1* expression. The indicated strains were analyzed. In all cases, littermate control mice were floxed for the indicated SIK mutant allele but negative for the Six2-Cre transgene. (**A**) Serum calcium, (**B**) phosphorus, (**C**) 1,25-vitamin D, and (**D**) PTH were measured, (**E**) along with kidney RNA for *Cyp27b1* and (**F**) the 1,25-vitamin D target gene *Cyp24a1*. Only SIK2/SIK3 double-KO mice showed a comparable phenotype as Six2-Cre;*Sik1*^fl/+^; *Sik2*^fl/fl^; *Sik3*^fl/fl^ animals, with increased *Cyp27b1*, mild hypercalcemia, 1,25-vitamin D, and suppressed PTH. Data are shown as the mean ± SD, and each dot represents an individual mouse. Student’s *t* test was performed against littermate controls for each genotype, and *P* values of less than 0.05 are shown. See also Supplemental Figure 10 for gDNA and mRNA gene deletion and urine data.

Next, we examined which SIK isoforms are responsible for these mineral metabolism effects. All combinations of SIK isoforms were deleted using Six2-Cre mice (Figure 6 and Supplemental Figure 10, A–C). SIK deletion was deemed adequate in mice, with the exception, for reasons that we do not currently understand, of Six2-Cre;*Sik1*^fl/fl^;*Sik2*^fl/fl^ mice which were therefore excluded from further analysis. Similar to Six2-Cre*;Sik1*^fl/+^;*Sik2*^fl/fl^;*Sik3*^fl/fl^ mice, only Six2-Cre;*Sik2*^fl/fl^;*Sik3*^fl/fl^ mice showed increased *Cyp27b1* expression and serum 1,25-vitamin D levels, mild (but statistically significant) hypercalcemia, and PTH suppression (Figure 6 and Supplemental Figure 10, D–H). Consistent with our results in Six2-Cre*;Sik1*^fl/+^;*Sik2*^fl/fl^;*Sik3*^fl/fl^ mice, Six2-Cre;*Sik2*^fl/fl^;*Sik3*^fl/fl^ animals also showed increased FGF23 levels and elevated bone turnover markers (Supplemental Figure 10, I–L). Furthermore, global inducible *Sik2* and *Sik3* deletion using Ubiquitin-Cre^ERt2^ also showed a similar mineral metabolism phenotype (Supplemental Figure 11). Taken together, these data demonstrate that SIK2 and SIK3 are the key isoforms responsible for *Cyp27b1* gene regulation downstream of renal PTH signaling.

### Renal SIKs control CRTC2 phosphorylation, nuclear localization, and Cyp27b1 enhancer binding.

**Figure 7:**
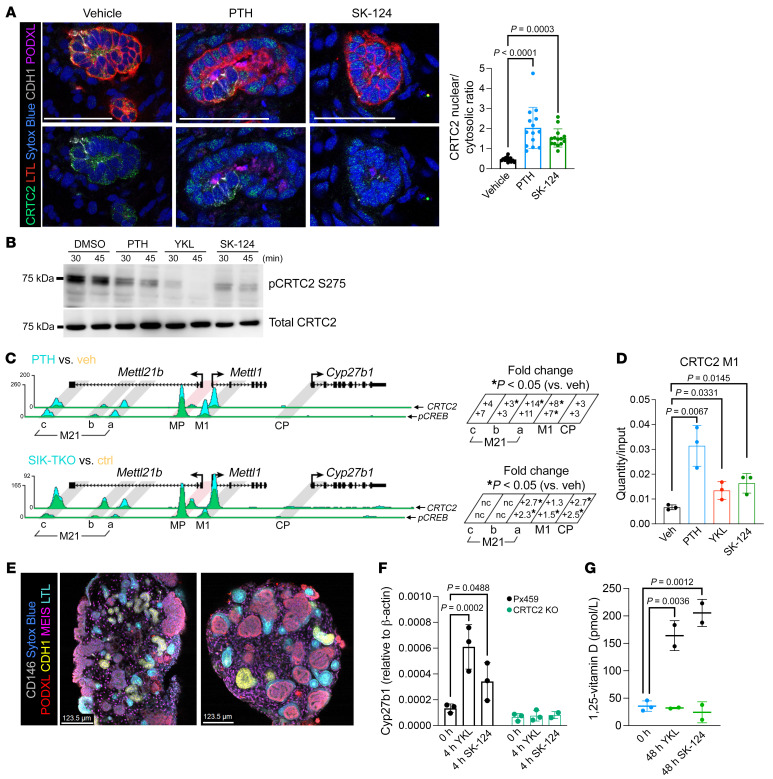
CRTC2 plays a crucial role in *Cyp27b1* transcriptional control downstream of PTH/SIK signaling. (**A**) Kidney organoids were treated with DMSO, PTH (242 nM), or SK-124 (10 μM) for 3 hours and immunostained to show CRTC2 nuclear translocation in Lotus Tetragonolobus Lectin–positive (LTL-positive) proximal tubules. Images on the top and bottom are identical with the exception that images on the lower row are shown without the LTL channel (LTL for proximal tubule; CDH1 for distal tubule; podocalyxin [PODXL] for podocytes; Sytox Blue as the nuclear dye). Scale bars: 50 μm. Quantification is shown in the histogram on the right, with *P* values from 1-way ANOVA followed by Tukey’s post hoc test. (**B**) Opossum kidney (OK) cells were treated for the indicated times with PTH (242 nM), YKL-05-099 (10 μM), and SK-124 (20 μM), followed by total and phospho-CRTC2 S275 immunoblotting. (**C**) Top: Mice were treated with PTH (230 ng/g, 30 min, i.p.) or vehicle followed by ChIP-Seq for CRTC2 or phospho-CREB (*n* = 3 per treatment). Bottom: Control and inducible/global SIK1/2/3 TKO mice (*n* = 3 per genotype) (see Figure 4) were treated with tamoxifen and sacrificed 48 hours later followed by kidney ChIP-Seq. Quantification of peak density is shown on the right for the various M21 enhancer subpeaks, along with the M1 enhancer and the Cyp27b1 promoter (CP). Fold change (versus vehicle) or peak density is shown, with asterisks indicating inducible binding of the indicated protein (CRTC2 on the top, pCREB on the bottom) at each site. (**D**) CRTC2 ChIP-qPCR using M1 enhancer primers was performed on kidneys from mice treated for 30 minutes as indicated. Recovered DNA relative to input samples is shown. (**E**) Representative images of Px459 and CRTC2-KO organoids show the development of nephron segments including proximal tubules by LTL staining. (MEIS for stromal cells; CDH1 for distal tubule; CD146 for vasculature; Sytox-Blue is for nucleic acid, staining all the cells.) Scale bars: 123.5 μm. (**F** and **G**) Cyp27b1 expression and 1,25-vitamin D secretion after SIK inhibitor treatment with YKL-05-099 (10 μM) and SK-124 (20 μM) in PX459 and CRTC2-KO kidney organoids are shown. Blue dots in **G** indicate the baseline 0-hour control for both YKL-05-099 and SK-124 drugs.

Next, we sought to understand how SIKs control *Cyp27b1* expression. The transcription factor CREB binds kidney-specific, PTH-responsive enhancers near the *Cyp27b1* gene (13). We focused on CRTC2 because members of the CRTC family are well-established SIK substrates that stimulate CREB activity (23), and CRTC2 was the most highly expressed isoform of CRTC family in kidney (Supplemental Figure 12, A and B, shows renal CRTC and SIK isoform gene expression counts). CRTC2 nuclear translocation occurs in human kidney organoids treated with either PTH or SIK inhibitors (Figure 7A). Moreover, SK-124 stimulated CRTC nuclear translocation in a heterologous subcellular localization assay (Supplemental Figure 13, A and B; EC_50_ 32 nM). Next, we measured phospho-CRTC2 protein abundance by immunoblotting in opossum kidney cells, a PTH-responsive proximal tubule cell line (51) (Supplemental Figure 12C). Treatment with both PTH and SIK inhibitors reduced CRTC2 phosphorylation (Figure 7B) and increased CRTC2 nuclear translocation (Supplemental Figure 13, C–E). To examine whether CRTC2 associates with *Cyp27b1* gene regulatory regions, CRTC2 ChIP-Seq was performed in mouse kidneys after acute PTH or SIK inhibitor injection. We observed significant increases in CRTC2 binding to both M21 and M1 *Cyp27b1* enhancers at early time points following acute PTH or SIK inhibitor treatment (Figure 7, C and D, genome browser and quantification tracks from ref. 52). Ubiquitin-Cre^ERt2^;*Sik1*^fl/fl^;*Sik2*^fl/fl^;*Sik3*^fl/fl^ mice also showed increased CRTC2 M1 enhancer binding (Figure 7C). Next, we examined the functional role of CRTC2 in human kidney organoids. Following CRISPR/Cas9-mediated genome editing, single-cell H9 clones with CRTC2 indel mutations that lead to absent CRTC2 protein were identified (Supplemental Figure 14) and used to generate kidney organoids. Control and CRTC2-deficient organoids showed comparable staining for proximal tubule, distal nephron, and podocyte markers (Figure 7E). Upon SIK inhibitor treatment, CRTC2-deficient organoids failed to increase *Cyp27b1* expression or 1,25-vitamin D production (Figure 7, F and G), demonstrating a key role of CRTC2 in SIK-mediated vitamin D gene regulation in human kidney organoids.

**Figure 8:**
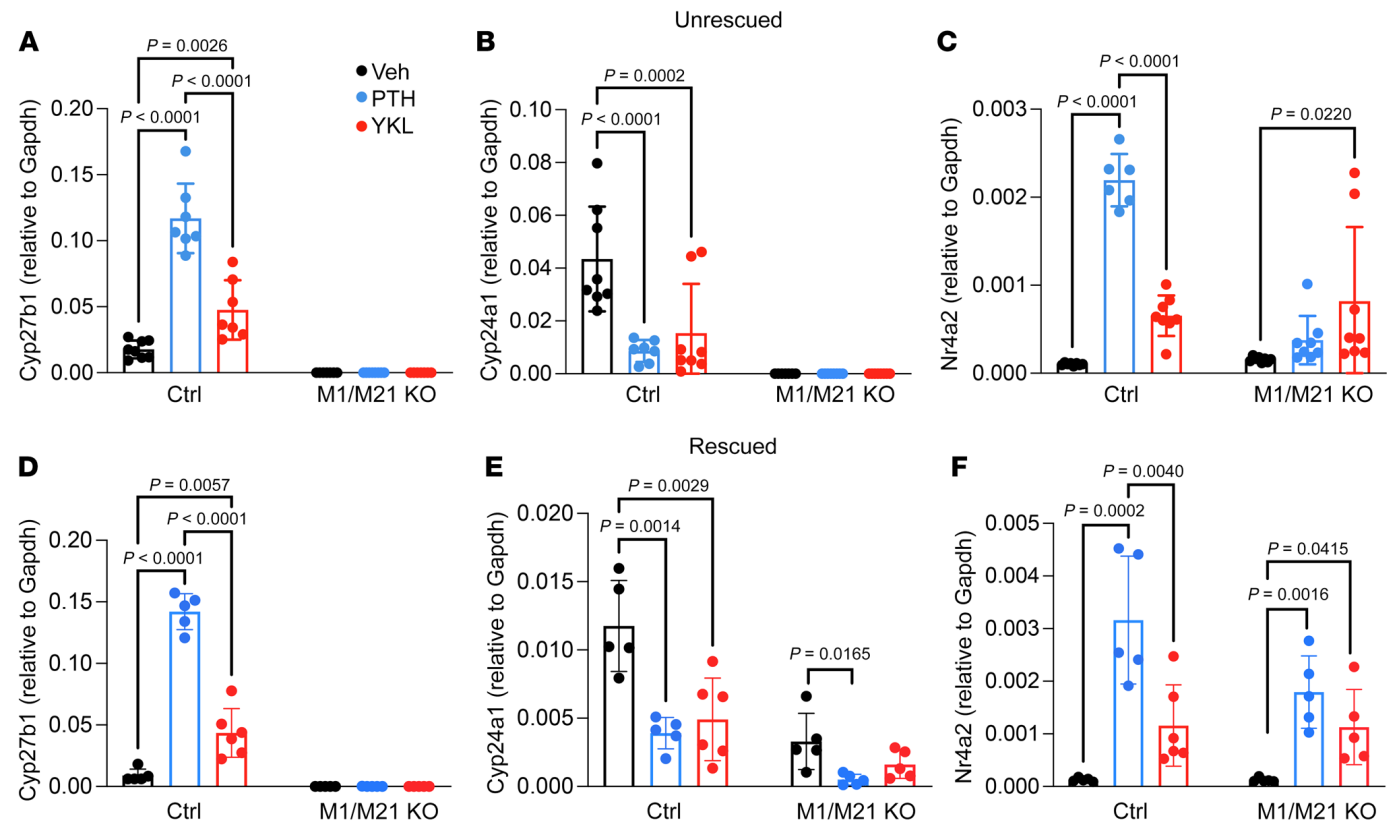
M1 and M21 *Cyp27b1* enhancers are required for SIK inhibitor–stimulated gene regulation. (**A**–**C**) Control and M1/M21 Cyp27b1 enhancer double-KO (DIKO) mice were treated with a single injection of vehicle, PTH (230 ng/g, 30 min, i.p.), or YKL-05-099 (45 mg/kg, 60 min, i.p.), and kidney RNA was isolated for RT-qPCR of the indicated gene. (**D**–**F**) Mice were fed a rescue diet to normalize calcium, phosphorous, and PTH for 16 weeks and then treated with a single dose of Veh, PTH, or YKL-05-099, followed by kidney RNA isolation and RT-qPCR. Data are shown as the mean ± SD, and each dot represents an individual mouse. One-way ANOVA within the same genotype followed by Tukey’s post hoc test was performed for statistical analysis. Adjusted *P* values are shown.

To assess the functional role of *Cyp27b1* M1 and M21 enhancers in SIK inhibitor–induced gene regulation in vivo, M1/M21-KO mice, which show defective basal and PTH-stimulated *Cyp27b1* expression (53), were treated with PTH and the SIK inhibitor YKL-05-099. As previously reported (53), these mice showed no PTH-induced *Cyp27b1* expression, while another target gene of PTH/SIK pathway, *Nr4a2* (24), showed preserved upregulation (Figure 8, A–C). Importantly, SIK inhibitor–induced *Cyp27b1* upregulation also depended on the presence of these CRTC2-binding enhancers. *Cyp24a1* mRNA in M1/M21-KO mice is undetectable due to absent 1,25-vitamin D levels and dramatic secondary hyperparathyroidism (53). Next, the same experiment was conducted in M1/M21-KO mice first fed a high-calcium/phosphorus rescue diet for 4 months to normalize serum calcium, phosphorus, and PTH levels (53). Importantly, “rescued” M1/M21-KO mice with detectable renal *Cyp24a1* expression also selectively lacked PTH- and SIK inhibitor–induced *Cyp27b1* expression (Figure 8, D–F). Together, these studies show that both PTH and SIK inhibitors signal through a pathway involving CRTC2, CREB, and the kidney-specific M1 and M21 enhancers to induce *Cyp27b1* expression and 1,25-vitamin D synthesis.

### SIK inhibitor treatment increases 1,25-vitamin D in mice with CKD-MBD.

**Figure 9:**
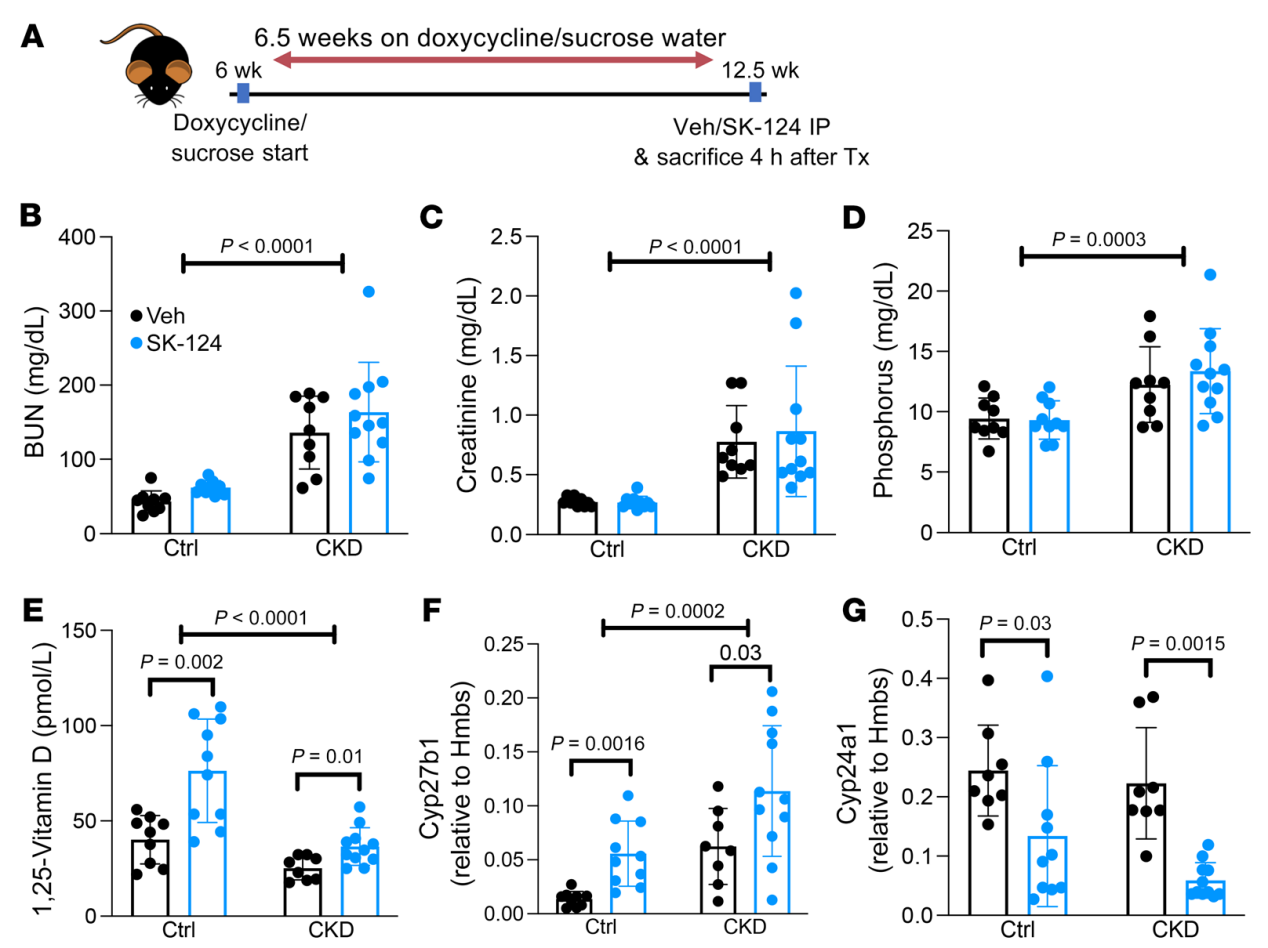
SIK inhibitor treatment increases renal *Cyp27b1* expression and 1,25-vitamin D levels in a mouse model of CKD-MBD. (**A**) Schema of study timeline. In inducible CTCF^podocyte–/–^ mice, doxycycline administration (to induce chronic kidney disease) causes podocyte loss and development of CKD-MBD compared with sucrose water administration (control). After 6.5 weeks of doxycycline or sucrose water, mice were treated with a single dose of either vehicle or SK-124 (40 mg/kg, i.p.), and sera were collected 4 hours later. (**B**–**E**) Serum parameters were measured in the indicated groups. (**F** and **G**) Kidney RNA was isolated for RT-qPCR. Data are shown as the mean ± SD, and each dot represents an individual mouse. Two-way ANOVA was performed, followed by post hoc *t* tests within each disease group (control group mice administered sucrose or CKD mice administered doxycycline) to determine if the SK-124 treatment effect was statistically significant.

In CKD-MBD, renal 1,25-vitamin D synthesis decreases despite secondary hyperparathyroidism (also known as PTH resistance or hyporesponsiveness) (54, 55). Elevated FGF23 levels also contribute to defective renal 1,25-vitamin D production (46). In addition, downregulation of PTH receptors could contribute to this complex pathophysiology (55). Thus, we hypothesized that targeting downstream signaling steps with SIK inhibitors might induce 1,25-vitamin D stimulation, even in the setting of early-stage kidney injury, by bypassing defects due to PTH receptor downregulation and high serum levels of FGF23. Doxycycline-inducible, podocyte-specific CTCF ablation mice (56) were selected for their gradual CKD development and clinically relevant CKD-MBD manifestations. After access to doxycycline-containing water for 6.5 weeks, a time point when proteinuria, defective renal function, and hyperparathyroidism with elevated FGF23 and low 1,25-vitamin D are present, mice were treated once with vehicle or SK-124 and euthanized 4 hours later (Figure 9A), a time point determined based on initial assessment of the kinetics of SK-124–induced *Cyp27b1* induction and serum 1,25-vitamin D increases in control and CKD mice (Supplemental Figure 15). The development of CKD-MBD was confirmed by the presence of high serum BUN, creatinine, and phosphate (Figure 9, B–D) as well as FGF23 and PTH levels (Supplemental Figure 16, A and B) without significant differences between the acute drug administration (vehicle versus SK-124) groups. Remarkably, acute SK-124 treatment stimulated 1,25-vitamin D production by increasing *Cyp27b1* and decreasing *Cyp24a1* expression in both control and CKD-MBD mice (Figure 9, E–G). These SIK inhibitor changes in *Cyp27b1* mRNA and 1,25-vitamin D levels occurred without changes in PTH, FGF23, serum and urine calcium, or urine phosphate at this 4-hour time point (Supplemental Figure 16). Overall, these findings demonstrate that SIK inhibitors can stimulate 1,25-vitamin D synthesis in CKD-MBD despite FGF23 excess.

## Discussion

Vitamin D is best known for its role in calcium homeostasis and bone health (57), but emerging studies have suggested immune-regulatory functions (58). Most circulating active vitamin D (1,25-vitamin D) is produced in the kidneys through the conversion of 25-vitamin D to 1,25-vitamin D under the control of PTH. In this study, we demonstrate that a signaling pathway involving SIK inhibition is responsible for renal PTH-induced vitamin D activation. Pharmacologic SIK inhibitors and genetic SIK ablation lead to increased renal *Cyp27b1* expression and increased circulating levels of 1,25-vitamin D. Analogous to how PTH regulates physiologically important target genes in mesenchymal lineage bone cells (24–26, 59), renal PTH signaling also uses SIK2 and SIK3 as central mediators to achieve its physiologic action in controlling vitamin D synthesis. Interestingly, these are the same SIK isoforms that control bone homeostasis (25). The genes for SIK2 and SIK3 are located in close proximity on the same chromosome in humans and mice (chromosome 11 in humans; chromosome 9 in mice), and the kinases are tonically “on” due to constitutive phosphorylation by the upstream kinase LKB1 (23). Given sequence similarity between SIK2 and SIK3, our data suggest that these 2 SIK isoforms might share redundant and compensatory roles in mineral metabolism both in kidney and bone. Although SIK1 and SIK3 are the most highly expressed SIK isoforms in kidney (Supplemental Figure 11A), our genetic results demonstrate a clear mineral metabolism phenotype in SIK2/SIK3 compound mutants that is not present in SIK1/SIK3 double-KO mice (Figure 6). Humans with homozygous loss-of-function SIK3 mutations show mild PTH-independent hypercalcemia (60), and common SIK3 variants are associated with blood calcium levels (61), findings that confirm the relevance of our findings in human mineral metabolism. However, the fact that combined SIK gene deletion was required to observe clear phenotypes in both bone and kidney suggests that functional redundancy between SIK isoforms is possible in multiple tissues (outside of the growth plate where SIK3 appears to play a unique role; refs. 25, 60) in mice.

Until now, the role of SIKs in normal renal physiology and kidney disease has not been well studied. SIK2 was identified as a protein with phosphorylation that was induced by vasopressin/Gαs/cAMP/PKA signaling in collecting duct cells by phospho-proteomics (62). Functional studies of the role of SIKs downstream of ADH action in the collecting duct for water homeostasis are currently lacking. Another report showed that in vivo renal SIK1 overexpression protects against the transition from initial renal injury into CKD by regulation of the WNT/β-catenin pathway (63). However, loss-of-function data regarding the roles of SIK isoforms in acute or chronic renal injury models remain to be determined. Here, we demonstrate that the major transcriptomic signature associated with SIK gene deletion involves the vitamin D pathway, a finding that is entirely consistent with our data demonstrating dysregulation of 1,25-vitamin D and calcium levels upon SIK gene deletion. Therefore, while it is possible that SIKs control physiologic functions in kidney other than mineral metabolism, our results highlight a major role for these kinases in vitamin D activation.

PTH has three major renal actions: (a) increasing *Cyp27b1* mRNA to increase 1,25-vitamin D production to enhance calcium absorption in gut (9), (b) stimulating calcium reabsorption in the distal tubule (64), and (c) increasing phosphate excretion in the proximal tubule via effects on Npt2a and Npt2c transporters (65). Our results identify a major role of SIKs in *Cyp27b1* mRNA regulation. Unlike PTH, which acutely lowers urine calcium clearance, we noted hypercalciuria in renal-specific SIK mutant mice. This finding is likely due to increased urinary calcium filtered load from 1,25-vitamin D–dependent bone resorption and gut calcium absorption. The absence of hypocalciuria in such animals suggests that SIKs may not directly participate in distal calcium reabsorption. The specific SIK2/SIK3 inhibitor SK-124 had no effects on blood or urinary phosphate levels, and these parameters were also “normal” in renal-specific SIK-KO mice. In theory, elevated 1,25-vitamin D in these animals might increase bone phosphate release and promote gut phosphate absorption, leading to challenges in interpreting the fact that urinary and blood phosphate levels were unchanged between control and renal SIK mutant animals. Nonetheless, the absence of overt hypophosphatemia or hyperphosphaturia (despite the caveat that our urine phosphate measurements were performed on “spot” samples rather than 24-hour urine collections) in such mice suggests that PTH-induced urinary phosphate excretion may also occur via a SIK-independent pathway, a model further supported by the lack of effects of SIK inhibitors on Npt2a localization and phosphate uptake in vitro (Supplemental Figure 8). The phosphaturic action of PTH mostly comes from a rapid posttranslational mechanism, in which the binding of Npt2a to its scaffold protein, NHERF1, is disrupted (66). In sum, while we cannot rule out minor effects of SIKs on distal calcium reabsorption and proximal urinary phosphate handling, these kinases play a dominant role in regulating 1,25-vitamin D physiology.

It has long been appreciated that renal 1,25-vitamin D synthesis is driven by PTH (31, 67, 68); furthermore, functional studies have suggested that PTH-stimulated *Cyp27b1* induction occurs in the proximal tubule (69), results supported by gene expression analyses performed several decades ago (70). Here we used scRNA-Seq to profile the cell type–specific renal response to PTH. In doing so, we noted that PTH-stimulated *Cyp27b1* induction occurs primarily in the S1 proximal convoluted tubule subsegment. In recent years, single-cell transcriptomic analyses have led to major advances in understanding of human and mouse renal physiology and disease (71, 72). This is the first study to our knowledge to examine the effects of PTH on renal gene expression at the single-cell transcriptomic level. Future studies will be needed to define renal changes that occur in response to chronic hyperparathyroidism in a cell-specific manner.

Renal control of vitamin D synthesis is achieved via hormonally regulated, tissue-specific *Cyp27b1* and *Cyp24a1* enhancers (13, 53, 73–75). The PTH-responsive Cyp27b1 M1 and M21 (named due to their intergenic location within the *Mettl1* and *Mettl21* genes) were initially identified due to renal-specific phospho-CREB binding. Here, we demonstrate that the CREB coactivator CRTC2, itself a SIK substrate, rapidly translocates into the nucleus and binds such enhancers in response to PTH and SIK inhibitor treatment. CRTC proteins stabilize protein/DNA interactions between CREB family transcription factors and target DNA elements and recruit other histone-modifying enzymes to specific genomic sites (76–78). CRTC2 ChIP-Seq in kidneys of PTH-treated mice shows substantial concordance between inducible genomic binding patterns between CRTC2 and CREB pS133 and gene expression changes (52). Since functional redundancy between CRTC isoforms has been reported (79, 80), studying the role of individual CRTC isoforms in 1,25-vitamin D metabolism, alone and in combination, will be of high interest. In addition, recent studies using altered PTH peptide ligands suggest that sustained endosomal signaling is needed to induce *Cyp27b1* mRNA (81); further studies are needed to determine whether endosome-generated cAMP pools are required for PKA-mediated SIK inactivation in proximal tubule cells.

Molecular studies of renal production of 1,25-vitamin D and the actions of PTH have been hampered by a lack of genuine proximal tubule cell lines. We used human embryonic stem cell–derived kidney organoids as a powerful tool (82). In contrast to 2D-cultured cell lines, kidney organoids contain heterogenous populations of kidney cells (83), similar to the first trimester human fetal kidney (84). PTH1R-expressing kidney organoids showed stimulation of Cyp27b1 expression by PTH and, importantly, a robust increase in 1,25-vitamin D protein in the media. FGF23, a hormone that lowers 1,25-vitamin D in vivo, also suppressed PTH inhibitor– and SIK inhibitor–induced 1,25-vitamin D production in this system. Thus, human kidney organoids represent a powerful model to explore physiologically relevant signaling events involved in mineral metabolism.

Fascinating differences are noted in skeletal and mineral metabolism phenotypes comparing bone-specific (25), ubiquitous (26), and renal-specific SIK gene deletion (reported here). Osteoblast/osteocyte-specific SIK2/SIK3 ablation leads to a dramatic increase in bone mass that closely resembles phenotypes observed when a constitutively active PTH receptor is expressed selectively in bone (25). Ubiquitous/inducible SIK2/SIK3 ablation leads to a comparable, yet somewhat less dramatic, high trabecular bone mass phenotype (26). Ubiquitous/inducible SIK1/SIK2/SIK3 deletion caused modest increases in trabecular bone mass, with dramatic 1,25-vitamin D–mediated hypercalcemia (Figure 4). Suppressing bone resorption with OPG-Fc partially rescued the accelerated mortality in these mice, indicating that hypercalcemia is a major (but perhaps not the only) cause of lethality upon inducible/global ablation of all 3 SIK isoforms. In contrast, renal-specific SIK ablation increased serum 1,25-vitamin D and calcium levels (Figures 5 and 6) and increased osteoclastic bone resorption and, therefore, reduced bone mass (Supplemental Figure 8). Taken together, these results suggest (a) that targeting the PTH/SIK pathway in only bone cells favors accrual of trabecular bone mass and (b) that the renal actions of the PTH/SIK pathway (at the level of stimulating 1,25-vitamin D synthesis) may oppose the bone anabolic effects of systemic PTH administration. These findings have important implications for emerging bone-targeting PTH delivery strategies (85–87).

In CKD-MBD, mineral ion derangements are linked to multiple complications (88), including morbidity and mortality (55, 89). CKD-MBD is a complex disease entity, with concurrent changes in phosphate, calcium, FGF23, Klotho, 1,25-vitamin D, and PTH all playing key roles (90, 91). Early in CKD, FGF23 increases to maintain normal blood phosphate levels, while suppressing 1,25-vitamin D. However, this compensatory mechanism ultimately fails, leading to hyperphosphatemia and hyperparathyroidism (90, 92). Because patients with advanced CKD show hyperparathyroidism as well as elevated levels of blood phosphate and low 1,25-vitamin D, the concept of PTH hyporesponsiveness has been long considered to be a component of CKD-MBD (55). Several cellular mechanisms have been proposed to account for this phenomenon (93–96). Until now, a lack of intracellular targets to mimic renal PTH action has limited the development of agents that increase 1,25-vitamin D synthesis in CKD. Our findings in the podocyte injury CKD model suggest that small-molecule SIK inhibitors bear potential for such a strategy. Further studies are needed to determine if SIK inhibitors increase 1,25-vitamin D levels of other CKD models with interstitial fibrosis and to assess the effect of long-term SIK inhibitor treatment on clinically relevant CKD-MBD endpoints, such as vascular calcification and renal osteodystrophy. In addition, these findings raise the possibility that oral SIK inhibitors might be useful agents for treatment of hypoparathyroidism.

These findings have important implications for the therapeutic development of SIK inhibitors. Our genetic studies suggest that simultaneous pharmacologic inhibition of all 3 SIK isoforms may not be tolerated but also may not be necessary at the level of bone and mineral metabolism. In addition, increases in blood calcium and 1,25-vitamin D could be used as biomarkers to monitor efficacy of selective SIK2/SIK3 inhibitors. In contrast, mice lacking SIK1 and SIK2 kinase activity show no obvious growth defects but altered cytokine production by macrophages (97), suggesting that distinct SIK isoforms selectively control inflammatory cytokine production versus mineral metabolism.

In addition to stimulating *Cyp27b1* mRNA, we note that pharmacologic SIK inhibitors also rapidly reduce expression of the catabolic vitamin D 24-hydroxylase *Cyp24a1*. By simultaneously increasing *Cyp27b1* and reducing *Cyp24a1*, the PTH/SIK pathway acutely reinforces the physiologic need to increase circulating 1,25-vitamin D levels. Future studies are needed to understand how PTH/SIK action suppresses *Cyp24a1* gene expression. In bone cells, PTH/SIK-regulated CRTC proteins stimulate CREB-dependent RANKL expression while PTH/SIK-regulated class IIa HDACs repress MEF2-driven sclerostin expression (24). Whether class IIa HDACs participate in PTH-mediated renal *Cyp24a1* suppression remains unknown.

In summary, this study highlights a crucial role for SIKs as mediators of PTH-induced vitamin D activation in the renal proximal tubule. In our model, SIKs are upstream of PTH-inducible, CRTC-driven Cyp27b1 expression. This model is built upon complementary pharmacologic, genetic, and genomic approaches in human and mouse systems. Human kidney organoids recapitulate hormonal control of 1,25-vitamin D synthesis and, thus, represent a powerful, clinically relevant model to interrogate signaling and gene regulatory mechanisms controlling vitamin D activation. Detailed understanding of the molecular cascade leading to PTH-induced 1,25-vitamin D generation leads to the possibility that small-molecule SIK inhibitors may be useful compounds to bypass PTH resistance and FGF23 excess to increase 1,25-vitamin D. Taken together, these findings further support our emerging model that SIKs participate in the physiologic actions of PTH. Future pharmacologic and genetic studies are needed to determine whether these PKA-regulated kinases participate in other endocrine GPCR signaling systems.

## Methods

Complete details regarding experimental procedures are provided in Supplemental Methods.

### Genetically modified mice.

C57BL/6J mice were obtained from The Jackson Laboratory. The following genetically modified mouse strains were used: *Sik1*-floxed mice (RRID MGI: 5648544; obtained in-house) (98), *Sik2*-floxed mice (RRID MGI: 5905012; obtained in-house) (99), and *Sik3*^tm1a^(EUCOMM)Hmgu mice (RRID MGI: 5085429) bred with PGK1 FLPo mice (The Jackson Laboratory, 011065); these strains were used to generate *Sik3*-floxed mice (25). Six2-Cre mice (43) were provided by Jordan Kreidberg (Boston Children’s Hospital, Boston, Massachusetts, USA), while Ubiquitin-Cre^ERt2^ mice (strain 007001) (100) and ROSA Ai14 TdTomato mice (strain 007914) (101) were obtained from The Jackson Laboratory. Cyp27b1 enhancer M1/M21 double-KO mice were as described in ref. 53 and housed at the University of Wisconsin — Madison.

Both male and female mice were used after verifying that there was no sex difference in mineral metabolism phenotype (see Results). At least 8 male or female mice were included for data analyses, and Cre-negative littermates were used as the controls.

### CTCF^podocyte–/–^ CKD model.

Inducible, podocyte-specific CTCF mutant mice (NPHS2-rtTA; TRE-Cre;*Ctcf*^fl/fl^) were as described in ref. 56. NPHS2-rtTA; TRE-Cre;*Ctcf*^fl/fl^ mice on doxycycline were used as CKD mice, while NPHS2-rtTA; TRE-Cre;*Ctcf*^fl/fl^ or *Ctcf*^fl/+^ mice on sucrose water and NPHS2-rtTA; TRE-Cre;*Ctcf*^+/+^ mice on doxycycline water were used as the controls. Both male and female mice were used as no sex difference was observed in CKD development or response to acute treatment with SK-124 compound. Mice were given either 5% sucrose (Sigma-Aldrich, S9378) only or 5% sucrose plus 0.4% Doxycycline (Sigma-Aldrich, D9891) in autoclaved RO drinking water in red, water bottles, starting at 6 weeks of age. Animals were fasted for 5 to 6 hours before euthanasia. After 6.5 weeks of special water treatment, mice were injected i.p. with either the vehicle (15% hydroxy-propyl β cyclodextrin; Sigma-Aldrich, H107) dissolved in ultra-pure water (Invitrogen, 10977023) or SK-124 dissolved in vehicle. After 4 hours, the animals were euthanized.

### Human kidney organoids.

H9 (WiCell) human pluripotent stem cells were maintained on hESC-qualified Geltrexcoated (Thermo Fisher Scientific) plates using StemFit Basic04 Complete (Ajinomoto Co. Inc.) as previously reported (33). hPSC lines were passaged weekly using Accutase (STEMCELL Technologies) for dissociation and Y27632 (Tocris) for adhesion. Directed differentiation of hPSCs into kidney organoids has been published elsewhere (102). For CRTC2 KO, the CRISPR/Cas9 system with Px459 backbone carrying gRNA targeting CRTC2 was used. More details can be found in Supplemental Methods.

### Data availability.

RNA-Seq data shown in Figure 1, C–E, have been deposited in the Gene Expression Omnibus (GEO; GSE207800). RNA-Seq data sets for Figure 1, H–K, and Figure 5, M and N, have been deposited in GEO (GSE207734). scRNA-Seq data have been deposited in GEO (GSE213316). ChIP-Seq data have been deposited in GEO (GSE206777). See complete unedited blots in the supplemental material.

### Statistics.

Data are expressed as means of biological repeats within a representative experiment ± SD. Statistical analyses between two groups were performed using an unpaired 2-tailed Student’s *t* test (GraphPad Prism 9.4.0). When more than two experimental groups were present, 1- or 2-way ANOVA analysis followed by post hoc test (indicated in figure legends) was performed. *P* values of less than 0.05 were considered as significant.

### Study approval.

All procedures involving animals were performed in accordance with guidelines issued by the Institutional Animal Care and Use Committees at the Center for Comparative Medicine at Massachusetts General Hospital under approved animal use protocols (2018N000023). All animals were housed at the Center for Comparative Medicine at Massachusetts General Hospital (21.9°C ± 0.8°C, 45% ± 15% humidity, and 12-hour light cycle of 7 am–7 pm). M1/M21 double-KO mouse studies were performed at the Research Animal Facility of the University of Wisconsin — Madison. These were reviewed and approved by the Research Animal Care and Use Committee of the University of Wisconsin — Madison.

## Author contributions

Research studies were designed by SHY, MBM, MT, TBS, RJX, CHA, DEF, NG, RM, AG, JWP, MM, and MNW. Experiments were conducted by SHY, MBM, MT, CZ, CDCA, KS, TS, NAB, SML, CHA, DJB, and NG. Data were acquired and analyzed by SHY, MBM, MT, CA, JSW, TBS, RJX, CHA, DJB, AA, RIS, IAR, NG, MM, and MNW. Reagents were provided by RB, MF, NG, RM, and AG. The manuscript was written by SHY, MM, and MNW. All authors edited and approved the manuscript.

## Supplementary Material

Supplemental data

Supplemental table 1

Supplemental table 2

Supplemental table 3

Supplemental table 4

Supplemental table 5

Supplemental table 6

Supplemental table 7

Supplemental table 8

Supplemental table 9

## Version 1 03/02/2023

In-Press Preview

## Version 2 05/01/2023

Electronic publication
