# Supplementary material for: A parathyroid hormone/salt-inducible kinase signaling axis controls renal vitamin D activation and organismal calcium homeostasis
Source: J Clin Invest. 2023 May 1;133(9):e163627. doi: 10.1172/JCI163627 (PMC10145948; doi:10.1172/JCI163627)

Supplemental Materials: contains supplemental methods, table legends, references, and figures/legends

Supplemental Methods

Genetically-modified mice – Global, Inducible SIK mutant mice

For studies with Ubiquitin-Cre^{ERt2} mice, tamoxifen (Sigma-Aldrich) was injected (1 mg, IP, Q48 hours, 3 injections total) in all animals including Cre-negative control littermates housed in cages with Pure-o'Cel bedding at 6 weeks of age. Next, mice were sacrificed 2 weeks (or 1-2 weeks for Ubiquitin-Cre^{ERt2}; *Sik1*$^{f/f}$;*Sik2*$^{f/f}$;*Sik3*$^{f/f}$ mice) following the first tamoxifen injection. Where indicated, tamoxifen was injected at 8 weeks of age and 200 µg OPG-Fc (200 µg/mouse, IP, provided by Amgen) dissolved in PBS was injected per mouse at the same time as the first tamoxifen injection, then once a week in the following weeks.

Compounds

Human PTH (1-34) was dissolved in 10 mM acetic acid for *in vivo* use and was further diluted in 0.1% BSA for *in vitro* use. YKL-05-099 was dissolved in 25 mM HCl in PBS (pH7.4) for *in vivo* use or in DMSO for *in vitro* use. SK-124 was dissolved in 15% 2-Hydroxypropyl-beta-cyclodextrin (H107; Sigma) in water for *in vivo* experiments and dissolved in DMSO for *in vitro* use. H89 Dihydrochloride (2910; Tocris), Bisindolylmaleimide I (203290; Sigma), YM-254890 (10-1590; Focus Biomolecules) were all dissolved in DMSO. For treating the organoids, recombinant human R179Q-FGF-23(25–251) (2604-FG-025; R&D Systems), resistant to furin cleavage and inactivation, was used after dissolving in 0.1% BSA in PBS. 25-hydroxycholecalciferol (H4014; Sigma) for the organoids was dissolved in 100% EtOH and aliquoted in amber tubes wrapped in parafilm and aluminum foil. All drugs were stored at -20°C, except for PTH, FGF23, and 25-OH-vitamin D, which were stored at -80°C.

Tissue Harvest

Fasting (5-6 hours) blood and spot urine samples were collected by cheek pouch bleeding and by pipetting urine collected on hydrophobic sand (Labsand; Braintree, MA). Kidneys were then immediately snap-frozen in liquid nitrogen for TRIzol (Life Technologies)-Chloroform (Fischer Scientific) based RNA isolation, or incubated in lysis buffer with proteinase K at 55°C for DNA isolation (DNeasy Blood and Tissue Kit; Qiagen, Hilden, Germany), or fixed in 10% formalin at 4°C for 24 hrs for histology.

RT-qPCR

cDNA was prepared using Primescript RT kit (Takara Inc), with 750 ng RNA per reaction. RT-qPCR was performed by using PerfeCa® SYBR® Green FAstMix® ROX (Quanta bio) in the StepOnePlus™ Real-time PCR System (Applied Biosystems). *Hmbs* was used as a control housekeeping gene for mouse samples, and *B-Actin* was used for organoids, relative expression was calculated ($2^{-deltaCT}$ method) and compared to the mean of control group if necessary. Primers used for RT-qPCR are listed in Supplemental Table 1.

Bulk RNA Sequencing

13-week-old C57BL6J male mice were injected with either 300 µg/kg body weight human PTH (1-34) (SQ) or 45 mg/kg YKL-05-099 (IP) and were sacrificed 1hr post treatment (n=8 per treatment). To determine the effects of SK-124, 8-week-old male C57BL6 mice were treated with Vehicle (Veh) or SK-124 (40 mg/kg, IP) and sacrificed 2-2.5 hrs later (n=6 per treatment). For Figure 5, 6-week old Six2-Cre; *Sik1*$^{f/+}$;*Sik2*$^{f/f}$;*Sik3*$^{f/f}$ and Cre negative littermates (n=4 per genotype) were sacrificed. RNA from whole mouse kidney was extracted by Trizol (Life Technology)/Chloroform based method, and submitted to BGI (Shenzhen, China) for RNA sequencing on BGISEQ500 platform. Sequencing reads were mapped to the mouse reference

genome (mm10/GRCm38) using STAR v.2.7.2b (1). Transcript abundances were calculated using RSEM v.1.3.0 (2). Differential gene expression analysis was performed using DESeq2 (3).

The degree of differential expression overlaps between two transcriptomic profiles (Suppl Figure 1C: PTH vs. YKL and Suppl Figure 1F: PTH vs. SK-124) was determined by Rank-Rank Hypergeometric Overlap (RRHO2) (4). Heatmaps generated using RRHO2 have the top right (both increasing) and bottom-left (both decreasing) quadrants, representing the concordant changes, while the top left and bottom right represent discordant overlap (opposite directional overlap between data sets). For each comparison, one-sided enrichment tests were used on $-\log_{10}$(nominal p values) with the default step size of 200 for each quadrant, and corrected Benjamini–Yekutieli p values were calculated. Heatmap (Figure 1J) was made using the ggplot2/tidyverse package (https://ggplot2.tidyverse.org) by calculating the log2FoldChange value of individual treatment library normalized to the averaged value of vehicles. Differentially expressed genes (DEGs) were selected if FDR (p-adjusted) < 0.05 and FC≥2 for pharmacologic studies, or FC>1.5 for Six2-Cre SIK mutant mouse kidney RNAs. Analysis of gene ontology enrichment was performed using Enrichr on DEGs (https://amp.pharm.mssm.edu/Enrichr/), and Venn diagrams were produced on this website (http://barc.wi.mit.edu/tools/venn/).

Histology

Kidneys were fixed in 10% buffered formalin for 24 hours prior to paraffin processing. Five µm sections were then subjected to standard H&E staining for basic histomorphology observation. Tibiae were fixed in 10% buffered formalin for 3-4 days, then decalcified in 15% EDTA for 2 weeks at room temperature for paraffin processing. For TRAP staining, 5 µm sections were first incubated in 0.2M acetate buffer (pH 5.0), then stained in the solution containing Napthol AS-MX Phosphate (0.5 mg/mL) and Fast Red TR Salt (1.1 mg/mL) in 0.2M acetate buffer, and

counterstained in Fast Green. The analysis was done in trabecular bone, approximately 200 μm away from the growth plate and cortical bone shaft, using Osteomeasure software (Osteometrics Inc, Decatur, GA, USA), to count TRAP positive osteoclasts, and the results are shown according to the standardized nomenclature (5). To visualize TdTomato expression in various tissues, tissues were submerged in 30% Sucrose, and embedded in O.C.T. Compound (4585, Fisher HealthCare), and cryo-sectioned. 10 μm sections were stained with DAPI (1:1000, D1306, Invitrogen) and imaged with Nanozoomer 2.0HT Slide Scanner (Hamamatsu Photonics).

Flow Cytometry

For Suppl Figure 4A-B, mice were subjected to intracardiac perfusion with 20mL cold PBS, and one kidney was dissected. The renal capsule was removed and half of the kidney was minced on petri dish in protease solution (2.5 mg/ml collagenase, type 2 (Worthington), 5 mM $CaCl_2$, 7.5 mg/ml Bacillus Licheniformis protease (Creative Enzymes), 125U/ml DNAse (Worthington) in DPBS). The sample was transferred to an Eppendorf tube and titrated every 5 minutes for 20-30 minutes. The reaction was terminated with 20% FBS, then the sample was filtered through 100 μm filter, centrifuged, and resuspended in the buffer with 0.5% BSA and 2mM EDTA. After removing red blood cells with Red Blood Cell Lysing Buffer Hybri-Max (R7767, Sigma), the cells were resuspended in FACS buffer (2% FBS in PBS) and analyzed on Attune NxT Flow Cytometer (ThermoFisher Scientific) for TdTomato expression. For Figure 5A-C, mice were perfused with 20ml cold PBS, then ½ of a kidney was quickly placed in cold RPMI media with 2% FBS on ice. After mincing with razor blade, the pieces were transferred to 1X Liberase TH (Millipore Sigma) dissociation medium and subjected to single cell dissociation as previously described except for glomeruli enrichment (6). Using FACSAria at MGH Wellman Center for Photomedicine, tdTomato positive and negative cells were sorted in cell lysis buffer and RT-qPCR was performed after RNA isolation.

Serum and Urine Assays

All serum and urine measurements were performed using commercial bioassay detection reagents listed in Supplemental Table 2, following the manufacturers' instructions. Urine calcium and phosphate were normalized to urine creatinine. Vitamin D metabolites were measured by LC-MS/MS as previously reported (7).

Human embryonic stem cell culture and kidney organoid generation

H9 (WiCell) human pluripotent stem cells were maintained on hESC-qualified Geltrexcoated (Thermo Fisher Scientific) plates using StemFit Basic04 Complete (Ajinomoto Co. Inc.) as previously reported (8). hPSC lines were passaged weekly using Accutase (STEMCELL Technologies) for dissociation and Y27632 (Tocris) for adhesion. Directed differentiation of hPSCs into kidney organoids has been published elsewhere (9) . Briefly, hPSCs were differentiated into metanephric mesenchyme cells on day 8 by a three-step directed differentiation protocol. Metanephric mesenchyme was transferred into suspension culture in 96-well ultralow adhesion plates (Corning) and further differentiated into kidney organoids, as previously reported (8). Quality control measures of SIX2 immunostaining on day 8 of differentiation and CDH1, LTL, PODXL, CD146, MEIS1/2/3, and SYTOX-blue staining on day 21 were performed on all batches of kidney organoids to limit batch-to-batch heterogeneity. Kidney organoids were maintained in 200 μl of basal media consisting of Advanced RPMI (Thermo Fisher Scientific) and GlutaMAX (Thermo Fisher Scientific) in 96-well plates, as previously reported. Media changes were conducted three times weekly with removal of 90 μl and addition of 100 μl of fresh basal media.

PTH-TMR Staining

On differentiation day 46, live organoids were incubated with LTL-Fluorescein (Vector labs, FL-1321-2, DF 1:200) in the presence and absence of 100 nM tetramethylrhodamine-labeled PTH (PTH-TMR) for a duration of 1 hour. Treated kidney organoids were then mounted to glass slides with cover slips and imaged using a Nikon C1q confocal microscope. Image rendering was performed using the freely available Nikon EZ-C1 viewer software.

Visualization of CRTC2, pPKA substrates, and Npt2a on kidney organoids

Day 28 kidney organoids were treated with either vehicle, PTH, or SK-124 for a duration of 30 minutes or 3 hours. Following the treatment, the organoids were collected and immediately fixed in 4% PFA in PBS for 1 hour at room temperature, submerged in 30% sucrose overnight at 4°C, then embedded in O.C.T. Compound (4585, Fisher HealthCare). Subsequently, 10-μm frozen sections were prepared via cryo-sectioning by a cryostat. After 1hr blocking at room temperature, the sections were treated overnight at 4°C with primary antibodies in antibody dilution buffer (ADB) with biotinylated LTL (1:200, B-1325, Vector), rat anti-CDH1 (1:500, ab11512, Abcam), goat anti-PODXL (1:500, AF1658, R&D Systems), as well as the antibody of interest, either rabbit anti-CRTC2 (1:200, HPA028465, Sigma), rabbit phospho-(Ser/Thr) PKA substrate (1:100, 9621, Cell Signaling Technology) or rabbit anti-SLC34A1 (Npt2a) (1:100, NBP2-13328, Novus Biologicals) antibodies. For the optimum labeling of LTL by biotin/streptavidin interaction, the Streptavidin/Biotin Blocking Kit (SP-2002, Vector Laboratories) was applied according to the manufacturer's instructions. After washing, the samples were stained for 1 hour at RT with the nuclear dye-SYTOX Blue (1:500, S11348, Invitrogen) and the corresponding secondary antibodies in ADB such as streptavidin-AF750 (1:500, S21384, Invitrogen), donkey anti-rat IgG-AF488 (1:500, A21208, Invitrogen), donkey anti-goat IgG-AF647 (1:500, A21447, Invitrogen), and donkey anti-rabbit IgG-AF594 (1:500, ab150076, Abcam) conjugate antibodies, and slides were mounted with Vectashield antifade mounting medium (H1000, Vector Laboratories). Finally, the

images were captured by the STELLARIS 8 confocal microscope (Leica Microsystems) using 25X water immersion objective lens. For the quantification of pPKA substrates and CRTC2 signal intensity across the $CDH1^{-}LTL^{+}$ proximal tubules, line profile and histogram analyses were performed through the multiple proximal tubule segments by the Leica Application Suite X (LAS X) software platform. The CRTC2 intensity of both nuclear (Sytox $blue^{+}$ area) and cytoplasmic (perinuclear Sytox $blue^{-}$ area surrounded by LTL^{+} membrane) regions was collected from the histograms to calculate the nuclear/cytoplasmic CRTC2 ratio of the proximal tubules indicating the nuclear translocation of CRTC2 from the cytoplasm.

Organoid analysis: RNA extraction and serum 1,25-vitamin D measurement

Day 25-28 organoids were used for experiments. To extract RNA, organoids were collected using pipetting with autoclaved bore tips and were subjected to Trizol-Chloroform RNA extraction method. Due to its size and yield, 3-4 organoids were pooled together for one RNA sample, and GlycoBlue (AM9515, ThermoFisher) was added for better visibility and yield. To measure 1,25-vitamin D in culture media, the organoids were first put in reduced volume (50 µl) of media containing 500 nM 25-(OH)-vitamin D, to concentrate 1,25-$(OH)_2$-vitamin D. Similarly, 3 wells were pooled together for one sample for 1,25-vitamin D EIA.

CRISPR/Cas9 mutant generation in human pluripotent stem cells

CRTC2 knock-out lines of human pluripotent stem cells (hPSCs) were generated as previously described (10). Briefly, we cultured H9 (WiCell) hPSCs on 6-well plates coated with hESC-qualified Geltrex (Thermo Fisher Scientific) in StemFit Basic04 Complete Type (SF04C, Ajinomoto Co Inc.) until they reach 70% confluency. Then by using LipofectamineTM 3000 reagent, we transfected cells with 2.5 µg of Px459 or Px459 carrying gRNA against CRTC2. After incubation at 37°C for 24 hours, we removed the transfection reagents and selected transfected cells by using 1 µg/ml of puromycin in SF04C supplemented with 10 µM Y27632

(Tocris) overnight. After puromycin selection, cell expansion was supported by Y27632 until small colonies were formed for a few days, and then cells were maintained for another week without Y27632.

To obtain a clonal population, we expanded and replated puromycin selected cells onto 96-well flat bottom plates (Corning) at 1 cell/well as previously described (10). In brief, cells were dissociated by Accutase (STEMCELL Technologies) and resuspended in SF04C with 10μM Y27632 at 100 cells/10 ml. Then we put 100 μl of cell suspension to each well of 96-well plates coated with Geltrex. After culturing cells at 37°C for 3 days, single cell colonies were observed in approximately 20% of wells. Then we removed the Y27632 and expanded cells for another 4 days. Next, we disassociated each colony and plated them in two 24-well plates with each well consisting of 500 μl of SF04C with 10 μM Y27632. After one-week growing, *CRTC2* mutation was confirmed by DNA CRISPR sequencing (Massachusetts General Hospital Center for Computational and Integrative Biology DNA Core).

GFP-CRTC3 nuclear translocation assay

Mel624 cells were a gift from Dr. Cassian Yee. Cells were transduced with pCW-57.1 (Addgene plasmid 41393) Cas9-P2A-CRTC3-GFP and subcloned to homogenize expression and induction efficiency (Addgene 196674). The resulting cells (Mel624 CRTC3-GFP Dox-ON 'Clone 2') were plated onto glass coverslips in 12-well plates and treated the next day with 500 ng/ml doxycycline for 48 hr before SIK inhibitor treatment. After 120-minute SIK inhibitor treatment, coverslips were washed with PBS and fixed in 4% PFA in PBS for 15 minutes on ice. After fixation, cells were rewashed before incubation for 15 minutes with 5 μg/mL AlexaFluor647 conjugated wheat germ agglutinin (Invitrogen, W32466) in PBS. After another PBS wash, the wheat germ agglutinin counterstain was fixed with 4% PFA in PBS for 15 minutes. Coverslips were mounted with DAPI-containing mounting media (VectaShield, H-1200-10).

Imaging was performed using a Nikon A1 confocal microscope. Under 40X objective, ten images were collected at focal points 3.75 μm apart per sample. Maximum intensity projections were generated using ImageJ v2.3.0/1.53f (11). Image segmentation was performed with CellPose v2.0, using the wheat germ agglutinin signal as channel 1, the DAPI signal as channel 2, the "cyto2" model, and a 70 pixel diameter as inputs (12). Resulting segmentation masks and Z-projections were further analyzed with CellProfiler v4.2.1 (13). Cells touching the border of each image were excluded and overlap Pearson's Correlation between DAPI and GFP channels were calculated for the remaining cells. On average, 122 cells were quantified per image, with no less than 68 cells analyzed for any one sample. The mean correlation between all cells within a sample are presented.

Immunoblotting

Cells were washed with cold PBS then scraped into TNT buffer (Tris-NaCl-Tween buffer, 20mM Tris-HCl pH 8, 200 mM NaCl, 0.5% Triton X-100 containing 1X-protease inhibitor cocktail (Bimake Inc. 100X, Cat. # B14001), 1 mM DTT, 1 mM NaF, 1 mM Vanadate (New England Biolabs)). Next, samples were vortexed for 1 minute and centrifuged at 14,000g for 6 minutes at 4 deg, and the supernatant was collected. Subcellular fractionation samples were prepared as previously described (14). Briefly, cells were collected and resuspended in hypotonic buffer (20 mM HEPES, 10 mM KCl, 1 mM $MgCl_2$, 0.1% Triton X-100, 5% glycerol supplemented with DTT, protease inhibitors, and phosphatase inhibitors) for 5 minutes on ice. After centrifuging at 5000g for 5 minutes at 4°C, the supernatant was collected as the cytosolic lysate. The nuclear protein pellet was vortexed in hypertonic buffer with 400 mM NaCl, and the supernatant was collected after centrifuge for the nuclear lysate.

For immunoblotting, the lysates were separated by SDS-PAGE, and proteins were transferred to the nitrocellulose membranes. Then, the membranes were blocked in 5% milk in tris-buffered saline plus 0.05% Tween-20 (TBST) and incubated with primary antibody in 5% BSA-TBST overnight at 4°C. Next day, after washing, membranes were incubated with HRP-coupled secondary antibody (1:2500, Cell Signaling Technology, 7074S), and signals were detected with SuperSignal West Femto Maximum Sensitivity Substrate (Thermo scientific) using Azure Biosystems C600 Imager. The antibodies used are: pPKA substrates (1:1000, Cell Signaling Technology, 100G7E), Total CRTC2 (1:1000, EMD Millipore, ST1099), Β-tubulin (1:500, Cell Signaling Technology, 5346), and Sp-1 (1:1000, EMD Millipore, 07-645). The CRTC2 pS275 (1:1000) antibody was as described in (15). Immunoblots were quantified on ImageJ according to the instruction (http://www.navbo.info/DensitometricAnalysys-NIHimage.pdf).

ChIP-Sequencing

ChIP-seq results shown (Figure 7) are from (16). The following reagents were used for *in vivo* injections: Parathyroid Hormone (PTH, 1-84 human) was obtained from Bachem (H-1370.0100 Torrence, CA), YKL-05-099 ((17)), and SK-124 (18). Diluent for the SK-124 was 15% HPBCD (hydroxypropyl β-cyclodextrin, Sigma, cat # H107) in sterile water. Antibodies used for ChIP-seq analysis were phosphorylated-CREB (Ser133) (pCREB, 06-519, lot # 3460466) purchased from EMD Millipore (Burlington, MA) and CRTC2 (A300-737A, lot# 1), purchased from Bethyl Laboratories (Montgomery, TX). Traditional genotyping PCR was completed with GoTaq (Promega, Madison, WI) and all real-time qPCR was completed with the StepOnePlus using TaqMan for gene expression assays (Applied Biosystems, Foster City, CA). Primers were obtained from IDT (Coralville, IA).

Chromatin Immunoprecipitation (ChIP) was performed using antibodies listed in Reagents. ChIP was performed as described previously with several modifications (19, 20). The isolated DNA

(or Input DNA acquired prior to precipitation) was then validated by quantitative real time PCR (qPCR) and further prepared for ChIP-seq analysis. *Cyp27b1* M1 enhancer ChIP PCR primers were 5'-TCTACTCTGGGTCTGTGGCCTT and 5'-AGCTAGACAGAACAACCGGGG. ChIP-seq libraries were prepared as previously described (20, 21) with the following exceptions: ChIP-seq libraries were prepared using the NEBNext Ultra II DNA kit (NEB, #E7645S) with the NEBNext Multiplex Oligos for Illumina (NEB, #E6440S) according to the manufacturer's protocols. Libraries were submitted to the University of Wisconsin – Madison Biotechnology Center's DNA Sequencing Facility (Research Resource Identifier – RRID:SCR_017759). Libraries were sequenced on a NovaSeq 6000. Paired end, 250 bp sequencing with a target of 20+ million reads was performed. Data was processed from NovaSeq 6000 with bcl2fastq. For ChIP-seq fold changes (indicated in each figure), ChIP-seq tag density was evaluated at each enhancer region compared to the tag density for the Vehicle treatment. These calculations were processed by HOMER, EdgeR, and DESeq2 (22-24).

Cyp27b1 enhancer mouse knockout studies

8-9 week-old C57BL/6 mice (The Jackson Laboratory, Bar Harbor, ME) were housed in high density ventilated caging in the Animal Research Facility of University of Wisconsin-Madison under 12-hour light/dark cycles at 72°F and 45% humidity. All mice were maintained on a standard rodent chow diet (5008, Lab Diet, St. Louis, MO). All experiments and tissue collections were performed in the procedure rooms in the Research Animal Facility of University of Wisconsin-Madison. All animal studies were reviewed and approved by the Research Animal Care and Use Committee of University of Wisconsin-Madison under protocol A005478. Both WT and M1/M21-DIKO (25) animals were subjected to intraperitoneal injection of 230 mcg/kg PTH (1–84) (in phosphate buffered saline (PBS)), 30 mg/kg YKL-05-099 (PBS + 25mM HCl), 40 mg/kg SK-124 (15% HPBCD (hydroxypropyl β-cyclodextrin)), or vehicle (EtOH, PBS, or HPBCD). Animals were sacrificed and tissues collected at times indicated in each legend for

ChIP and gene expression. Unless otherwise indicated, all experiments were conducted with equal numbers of males and females (n ≥ 6). Data were reported as mixed, as no differences were found between sexes.

MicroCT analysis

Left femurs from 6-week old male Six2-Cre; *Sik1*$^{f/+}$;*Sik2*$^{f/f}$;*Sik3*$^{f/f}$ mice were used for MicroCT analysis. A high-resolution desktop micro-tomographic imaging system (µCT40, Scanco Medical AG, Brüttisellen, Switzerland) was used to assess trabecular bone architecture in the distal femoral metaphysis and cortical bone morphology of the femoral mid-diaphysis. Scans were acquired using a 10 µm^3 isotropic voxel size, 70 kVP, 114 µA, 200 ms integration time, and were subjected to Gaussian filtration and segmentation. Image acquisition and analysis protocols adhered to the guidelines for the assessment of rodent bones using µCT (26). In the femur, trabecular bone microarchitecture was evaluated in a 1.5 mm (150 transverse slices) long region of the distal metaphysis beginning 200 µm superior to the peak of the growth plate and extending proximally. The trabecular bone regions were identified by manually contouring the endocortical region of the bone. A threshold of 400 mgHA/cm^3 was used to segment bone from soft tissue and the standard trabecular bone morphology script in the Scanco Evaluation Program was used to measure trabecular bone volume fraction (Tb.BV/TV, %), trabecular bone mineral density (Tb. BMD, mgHA/cm^3), trabecular thickness (Tb.Th, mm), trabecular number (Tb.N, mm^{-1}), trabecular separation (Tb.Sp, mm), connectivity density (Conn.D, 1/mm^3), and structural model index (SMI). Cortical bone was assessed in 50 transverse µCT slices (500 µm long region) at the femoral mid-diaphysis and the region included the entire outer most edge of the cortex. Cortical bone was segmented using a fixed threshold of 700 mgHA/cm^3 and the Scanco midshaft evaluation script was used to measure total cross-sectional area (Tt.Ar, mm^2), cortical bone area (Ct.Ar, mm^2), medullary area (Ma.Ar, mm^2), cortical bone area fraction

(Ct.Ar/Tt.Ar, %), cortical tissue mineral density (Ct.TMD, mgHA/cm^3), cortical thickness (Ct.Th, mm), as well as the maximum, minimum and polar moments of inertia (I_{max}, I_{min}, and J, mm^4).

Single cell-RNA-Seq

Mice were injected (i.p.) with either vehicle, hPTH (PTH 1-34, 300 µg/kg), or SK-124 (40 mg/kg) and sacrificed using CO_2 inhalation, 90 min or 2.5-3hr post injection, respectively. The animals were perfused with 20ml of cold PBS, then ½ of a kidney was quickly placed in cold RPMI media with 2% FBS on ice. After mincing with razor blade, the pieces were transferred to 1X Liberase TH (Millipore Sigma) dissociation medium and subjected to single cell dissociation as previously described except for glomeruli enrichment (6).

All scRNA-seq data were generated via 10X Genomics Chromium 3' droplet-based sequencing. The *CellRanger* toolkit (v6.0.2) (Genomics, n.d.) by 10X Genomics was used to 1) align the sequencing reads to the mouse reference transcriptome (mm10), 2) quantify gene expression, resulting in gene-by-cell UMI count matrices, and 3) aggregate all samples into a single feature-barcode matrix containing all the data for downstream analysis. All single cell data analysis was performed in the R software environment (v4.0.3) for statistical computing and graphics using the package Seurat (v4.1.1) (27). As an initial QC for all samples, only cells with more than 200 and less than 2500 genes were retained and no UMI cutoff for reads mapping was applied. Violin plots were used for visualization of the number of UMIs, genes and percent of mitochondrial reads (percent.mito) after these filters. After no technical batch effect was found, we proceeded to downstream processing steps and clustering of cells. We normalized and then log-transformed the UMI counts per cell using the *NormalizeData* function and default scale factor of 10,000. Next, data was scaled to assure different arrays have the same median value and dynamic range using the *ScaleData* function centering across all genes and regressing out *percent.mito*. Because the kidney has the second highest mitochondrial metabolic activity

among organs in the human body (28), we imposed a permissive threshold of 50% mitochondrial reads based on the canonical markers, both statistical and percentage of cell expression, confidence and PCT subsegments (S1, S2 and S3) clustering. We identified cell-to-cell high varying features of gene expression using the *FindVariableFeatures* function with default parameters. We computed the top 10 principal components (PCs) using the *RunPCA* function on the scaled expression matrix with only high variable genes.

For cell-types identification, we determined each cell type's nearest neighbor by an unsupervised clustering approach using the *FindNeighbors* function on all the top computed PCs. *FindNeighbors* builds an object with a shared nearest neighbor (SNN) graph on the gene expression level by calculating the neighborhood overlap between each cell and its nearest neighbors (*k.param*). Next, we found clusters using the *FindClusters* function with a resolution of 0.5. For visualization, cells were embedded using the Uniform manifold approximation and projection (UMAP) approach using the *RunUMAP* function with default parameters. We performed differential gene expression (DGE) analysis using the *FindAllMarkers* function to determine cluster-specific and high differentially expressed (DE) genes using Wilcoxon Rank-Sum test with False Discovery Rate (FDR) using the Benjamini-Hochberg procedure (Benjamani and Hochberg 1995 (29)). DGE was performed on genes expressed in at least 10% of cells (default *min.pct* argument) and filtered by positive expression with a *logfc.threshold* of 0.25. We built a collection of canonical kidney cell-specific markers from both experimental validation and literature (30-32). We derived cluster specific DE genes using the *FindAllMarkers* function for all clusters or cell-types with *logfc.threshold* parameter of 0.25. We visualized the top 10 markers predicted by the DGE analysis for each cluster on a heatmap. Next, we plotted the canonical markers for each respective cluster using the *DotPlot* function and added extra features using the *ggplot2* (Wickham 2016) R package. DGE analysis was performed within each cell-type using multiple comparisons (PTH/VEH, SK-124/VEH). The *FindMarkers* function

was used to run each comparison, respectively, with *logfc.threshold* of 0.25, *min.pct* of 0.1, and *wilcox* test parameter. The *wilcox* parameter identifies DE genes between the two groups of cells using a Wilcoxon Rank-Sum test with False Discovery Rate (FDR) using the Benjamini-Hochberg procedure (29). We summarized all DGE comparisons across all cell-types using a heatmap from the *geom_tile* function within the *ggplot2* package. Volcano plots for each comparison were made using the *geom_point* function within the *ggplot2* package.

In situ hybridization and PAS staining

Formalin-fixed paraffin embedded sections were used for *Cyp27b1* (503411, ACD) *in situ* hybridization according to the manufacturer instructions. Briefly, the sections were blocked with hydrogen peroxide for 10 minutes at room temperature, followed by antigen retrieval with Pepsin (R2283, Sigma-Aldrich) in humidified chamber in 40°C HybEZ oven. The *Cyp27b1* probe was added to the sections for 2 hours in the humidified chamber at 40°C. Then, the signal was amplified (322310, ACD) and detected using Brown DAB kit (322310, ACD). Serial sections were also subjected to Periodic acid and Schiff (PAS) staining (CP49819 and CP49820, Abcam) for overall kidney morphology and tubular structure identification using the manufacturer instructions.

P_i reuptake assay

P_i uptake assays were as performed in (33). Opossum Kidney (OK) epithelial cells were treated with PTH 1-34 or SK-124 at various doses for 4 hrs then incubated with ^{32}P (PerkinElmer Life Sciences) for 5 min. After rinsing, cells were lysed using 0.1N NaOH/1% Triton X-100 solution. Scintillation fluid was then added to the lysates, and total radioactivity was counted using a Beckman model LS600IC liquid scintillation analyzer. As a positive control, cells were treated with the Npt2a inhibitor PF-06869206 as in (34).

Supplemental Table Legends

Supplemental Table 1: Oligonucleotide primer sequences
Supplemental Table 2: Bioassay reagents used
Supplemental Table 3: Whole kidney RNA-seq, PTH versus vehicle
Supplemental Table 4: Whole kidney RNA-seq, YKL-05-099 versus vehicle
Supplemental Table 5: Whole kidney RNA-seq, SK-124 versus vehicle
Supplemental Table 6: scRNA-seq cell-specific DEGs, PTH versus vehicle
Supplemental Table 7: scRNA-seq cell-specific DEGs, SK-124 versus vehicle
Supplemental Table 8: Whole kidney RNA-seq, SIK mutant (Six2-Cre) versus control
Supplemental Table 9: MicroCT data, Six2-Cre SIK mutant versus control

Supplemental References

1. Dobin A, Davis CA, Schlesinger F, Drenkow J, Zaleski C, Jha S, et al. STAR: ultrafast universal RNA-seq aligner. Bioinformatics. 2013;29(1):15-21.
2. Li B, Dewey CN. RSEM: accurate transcript quantification from RNA-Seq data with or without a reference genome. BMC Bioinformatics. 2011;12:323.
3. Love MI, Huber W, Anders S. Moderated estimation of fold change and dispersion for RNA-seq data with DESeq2. Genome biology. 2014;15(12):550.
4. Cahill KM, Huo Z, Tseng GC, Logan RW, Seney ML. Improved identification of concordant and discordant gene expression signatures using an updated rank-rank hypergeometric overlap approach. Scientific reports. 2018;8(1):9588.
5. Dempster DW, Compston JE, Drezner MK, Glorieux FH, Kanis JA, Malluche H, et al. Standardized nomenclature, symbols, and units for bone histomorphometry: a 2012 update of the report of the ASBMR Histomorphometry Nomenclature Committee. Journal of bone and mineral research : the official journal of the American Society for Bone and Mineral Research. 2013;28(1):2-17.
6. Clark AR, Marshall J, Zhou Y, Montesinos MS, Chen H, Nguyen L, et al. Single-Cell Transcriptomics Reveal Disrupted Kidney Filter Cell-Cell Interactions after Early and Selective Podocyte Injury. Am J Pathol. 2022;192(2):281-94.
7. Meyer MB, Benkusky NA, Kaufmann M, Lee SM, Onal M, Jones G, et al. A kidney-specific genetic control module in mice governs endocrine regulation of the cytochrome P450 gene Cyp27b1 essential for vitamin D3 activation. J Biol Chem. 2017;292(42):17541-58.
8. Morizane R, Lam AQ, Freedman BS, Kishi S, Valerius MT, Bonventre JV. Nephron organoids derived from human pluripotent stem cells model kidney development and injury. Nature biotechnology. 2015;33(11):1193-200.
9. Morizane R, Bonventre JV. Generation of nephron progenitor cells and kidney organoids from human pluripotent stem cells. Nature protocols. 2017;12(1):195-207.
10. Gupta N, Susa K, Yoda Y, Bonventre JV, Valerius MT, Morizane R. CRISPR/Cas9-based Targeted Genome Editing for the Development of Monogenic Diseases Models with Human Pluripotent Stem Cells. Curr Protoc Stem Cell Biol. 2018;45(1):e50.
11. Schindelin J, Arganda-Carreras I, Frise E, Kaynig V, Longair M, Pietzsch T, et al. Fiji: an open-source platform for biological-image analysis. Nat Methods. 2012;9(7):676-82.
12. Stringer C, Wang T, Michaelos M, Pachitariu M. Cellpose: a generalist algorithm for cellular segmentation. Nat Methods. 2021;18(1):100-6.
13. Stirling DR, Swain-Bowden MJ, Lucas AM, Carpenter AE, Cimini BA, Goodman A. CellProfiler 4: improvements in speed, utility and usability. BMC Bioinformatics. 2021;22(1):433.
14. Sato T, Verma S, Andrade CDC, Omeara M, Campbell N, Wang JS, et al. A FAK/HDAC5 signaling axis controls osteocyte mechanotransduction. Nat Commun. 2020;11(1):3282.

15. Sato T, Verma S, Khatri A, Dean T, Goransson O, Gardella TJ, et al. Comparable Initial Engagement of Intracellular Signaling Pathways by Parathyroid Hormone Receptor Ligands Teriparatide, Abaloparatide, and Long-Acting PTH. JBMR Plus. 2021;5(5):e10441.
16. Meyer MB, Benkusky NA, Lee SM, Yoon SH, Mannstadt M, Wein MN, et al. Rapid genomic changes by mineralotropic hormones and kinase SIK inhibition drive coordinated renal Cyp27b1 and Cyp24a1 expression via CREB modules. J Biol Chem. 2022;298(11):102559.
17. Sundberg TB, Liang Y, Wu H, Choi HG, Kim ND, Sim T, et al. Development of Chemical Probes for Investigation of Salt-Inducible Kinase Function in Vivo. ACS Chem Biol. 2016;11(8):2105-11.
18. Sato T, Andrade CDC, Yoon SH, Zhao Y, Greenlee WJ, Weber PC, et al. Structure-based design of selective, orally available salt-inducible kinase inhibitors that stimulate bone formation in mice. Proceedings of the National Academy of Sciences of the United States of America. 2022;119(50):e2214396119.
19. Meyer MB, Zella LA, Nerenz RD, Pike JW. Characterizing early events associated with the activation of target genes by 1,25-dihydroxyvitamin D_3 in mouse kidney and intestine in vivo. J Biol Chem. 2007;282(31):22344-52.
20. Meyer MB, Benkusky NA, Kaufmann M, Lee SM, Onal M, Jones G, et al. A kidney-specific genetic control module in mice governs endocrine regulation of the cytochrome P450 gene. J Biol Chem. 2017;292(42):17541-58.
21. Meyer MB, Benkusky NA, Pike JW. The RUNX2 cistrome in osteoblasts: characterization, down-regulation following differentiation, and relationship to gene expression. J Biol Chem. 2014;289(23):16016-31.
22. Anders S, Huber W. Differential expression analysis for sequence count data. Genome biology. 2010;11(10):R106.
23. Robinson MD, McCarthy DJ, Smyth GK. edgeR: a Bioconductor package for differential expression analysis of digital gene expression data. Bioinformatics. 2010;26(1):139-40.
24. Homer-Bouthiette C, Doetschman T, Xiao L, Hurley MM. Knockout of nuclear high molecular weight FGF2 isoforms in mice modulates bone and phosphate homeostasis. J Biol Chem. 2014;289(52):36303-14.
25. Meyer MB, Benkusky NA, Kaufmann M, Lee SM, Redfield RR, Jones G, et al. Targeted genomic deletions identify diverse enhancer functions and generate a kidney-specific, endocrine-deficient *Cyp27b1* pseudo-null mouse. J Biol Chem. 2019;294(24):9518-35.
26. Bouxsein ML, Boyd SK, Christiansen BA, Guldberg RE, Jepsen KJ, Muller R. Guidelines for assessment of bone microstructure in rodents using micro-computed tomography. J Bone Miner Res. 2010;25(7):1468-86.
27. Hao Y, Hao S, Andersen-Nissen E, Mauck WM, 3rd, Zheng S, Butler A, et al. Integrated analysis of multimodal single-cell data. Cell. 2021;184(13):3573-87 e29.
28. Mercer TR, Neph S, Dinger ME, Crawford J, Smith MA, Shearwood AM, et al. The human mitochondrial transcriptome. Cell. 2011;146(4):645-58.
29. Benjamini Y, Hochberg Y. Controlling the False Discovery Rate: A Practical and Powerful Approach to Multiple Testing. Journal of the Royal Statistical Society. 1995;57(1):289-300.
30. Wilson PC, Wu H, Kirita Y, Uchimura K, Ledru N, Rennke HG, et al. The single-cell transcriptomic landscape of early human diabetic nephropathy. Proc Natl Acad Sci U S A. 2019;116(39):19619-25.
31. Muto Y, Wilson PC, Ledru N, Wu H, Dimke H, Waikar SS, et al. Single cell transcriptional and chromatin accessibility profiling redefine cellular heterogeneity in the adult human kidney. Nat Commun. 2021;12(1):2190.
32. Subramanian A, Sidhom EH, Emani M, Vernon K, Sahakian N, Zhou Y, et al. Single cell census of human kidney organoids shows reproducibility and diminished off-target cells after transplantation. Nat Commun. 2019;10(1):5462.

33. Jonsson KB, Mannstadt M, Miyauchi A, Yang IM, Stein G, Ljunggren O, et al. Extracts from tumors causing oncogenic osteomalacia inhibit phosphate uptake in opossum kidney cells. The Journal of endocrinology. 2001;169(3):613-20.
34. Clerin V, Saito H, Filipski KJ, Nguyen AH, Garren J, Kisucka J, et al. Selective pharmacological inhibition of the sodium-dependent phosphate cotransporter NPT2a promotes phosphate excretion. J Clin Invest. 2020;130(12):6510-22.

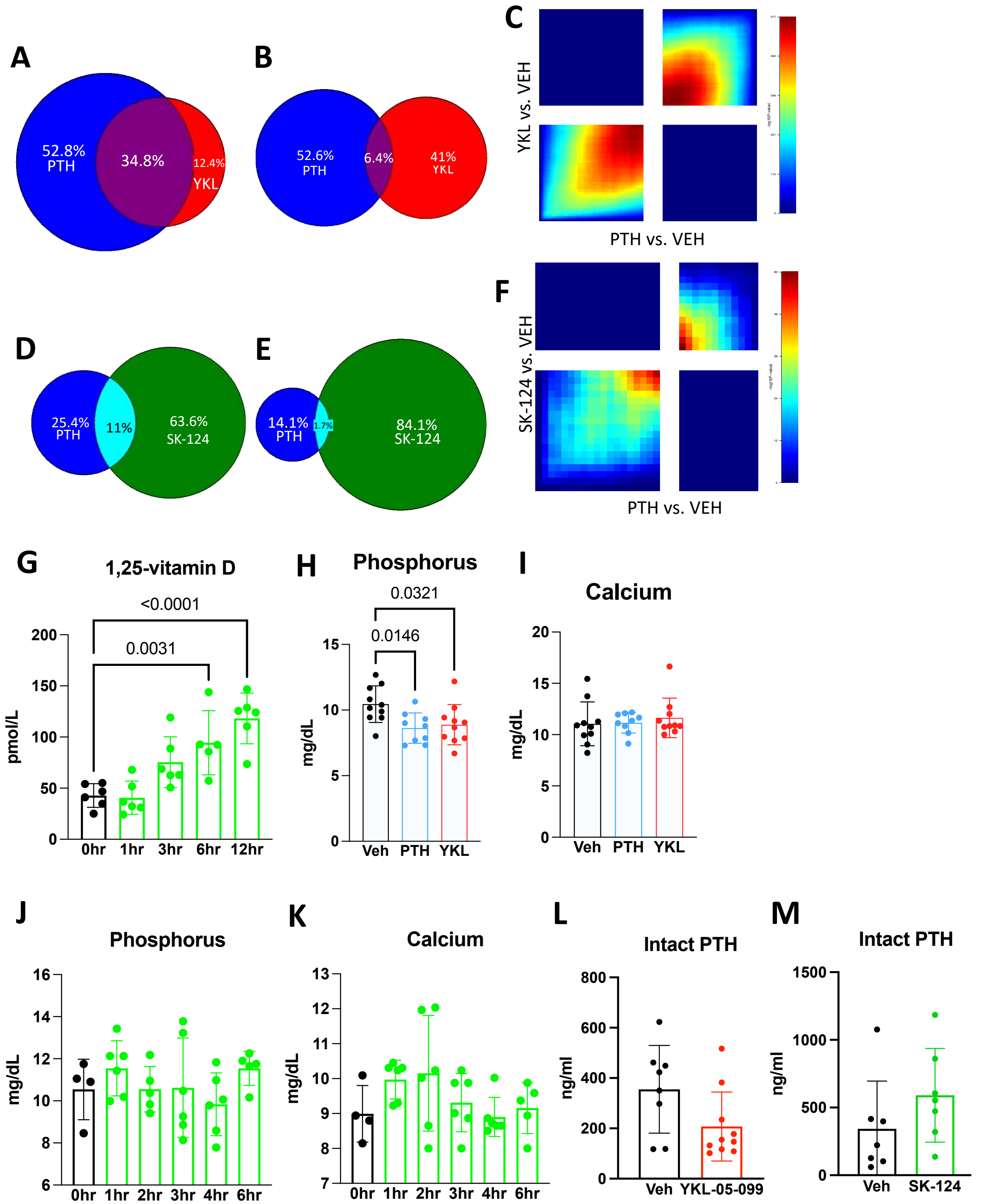

Supplemental Figure 1. Differentially upregulated (A, D) and downregulated (B, E) genes of PTH, YKL-05-099, and SK-124 datasets were compared in Venn diagrams (FC>2, FDR<0.05), and more detailed correlations between these treatment effects were demonstrated by RRHO2 (C, F). (G) 8-10-week-old control mice (iCTCF$^{pod-/-}$ mice not administered doxycycline) were treated with SK-124 (40 mg/kg, IP) and 1,25-vitamin D levels were measured at the indicated times. (H, I) 13-week-old C57BL6 mice were treated with vehicle, PTH (300 µg/kg, SC), YKL-05-099 (45 mg/kg, IP) and serum was collected 90 min (PTH) or 3 hours (YKL) later for calcium and phosphorus measurement. (J, K) 8-10-week-old control mice (iCTCF$^{pod-/-}$ mice not administered doxycycline) were treated with SK-124 (40 mg/kg, IP) and serum phosphorus and calcium were measured at the indicated times. Statistical comparisons (one-way ANOVA followed by post-hoc Tukey's test) versus vehicle-treated mice are shown. (L, M) Biointact PTH (1-84) levels were measured in 13-week C57BL6 wild type male mice treated with YKL-05-099 (45 mg/kg) (F) and 8-week C57BL6 wild type male mice treated with SK-124 (40 mg/kg). Student t-tests were performed and neither compound significantly affected circulating PTH levels.

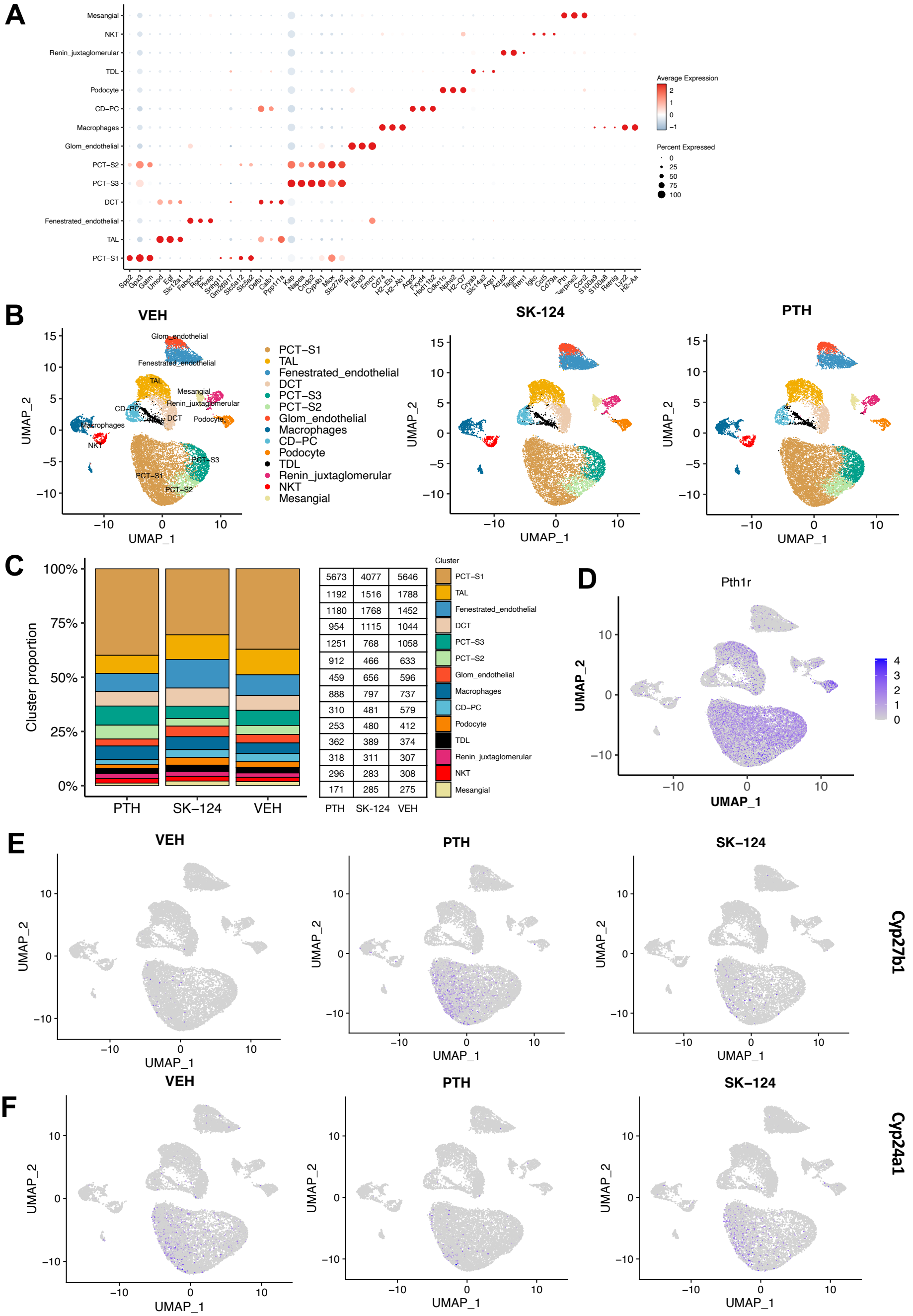

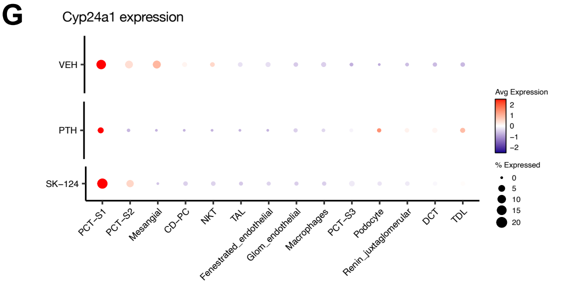

Supplemental Figure 2. (A) Dot plot showing marker genes used to identify each cluster. (B) UMAP representation of kidney scRNA-seq data by treatment groups. (C) Bar graph showing overall cluster cellularity by treatment groups. (D) *Pth1r* feature plot demonstrating high expression in proximal and distal epithelial cells and in podocytes. (E, F) Feature plots for *Cyp27b1* and *Cyp24a1* demonstrating gene expression changes in PCT cells. (G) Dot plot for *Cyp24a1* demonstrating that proximal tubule segment 1 is where PTH-induced *Cyp24a1* change is the most prominent. The color of the dots shows average gene expression level, while the size of the dots indicates the percentage of cells expressing the gene of interest in each cluster.

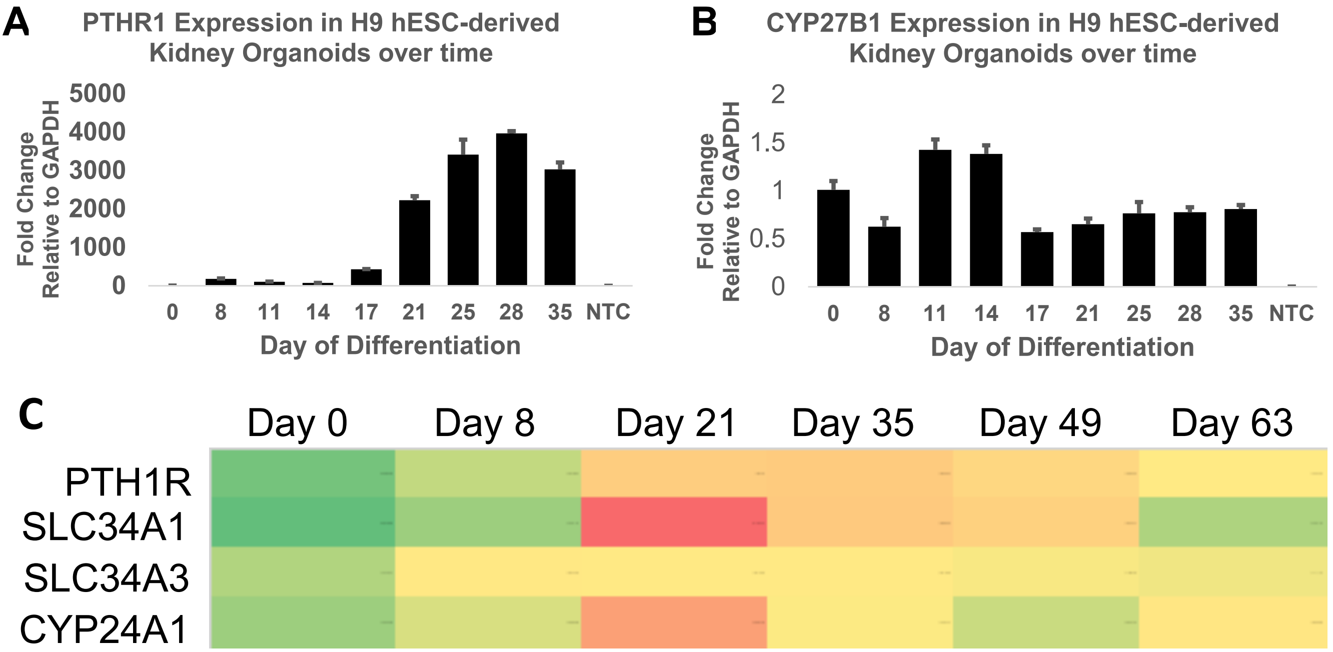

Supplemental Figure 3. (A, B) *Pth1r* and *Cyp27b1* expression in H9 human embryonic stem cell-derived kidney organoids over 35 days of differentiation by RT-qPCR. (C) RNA-sequencing data for *Pth1r*, *Slc34a1*, *Slc34a3*, and *Cyp24a1* at different time points throughout organoid differentiation.

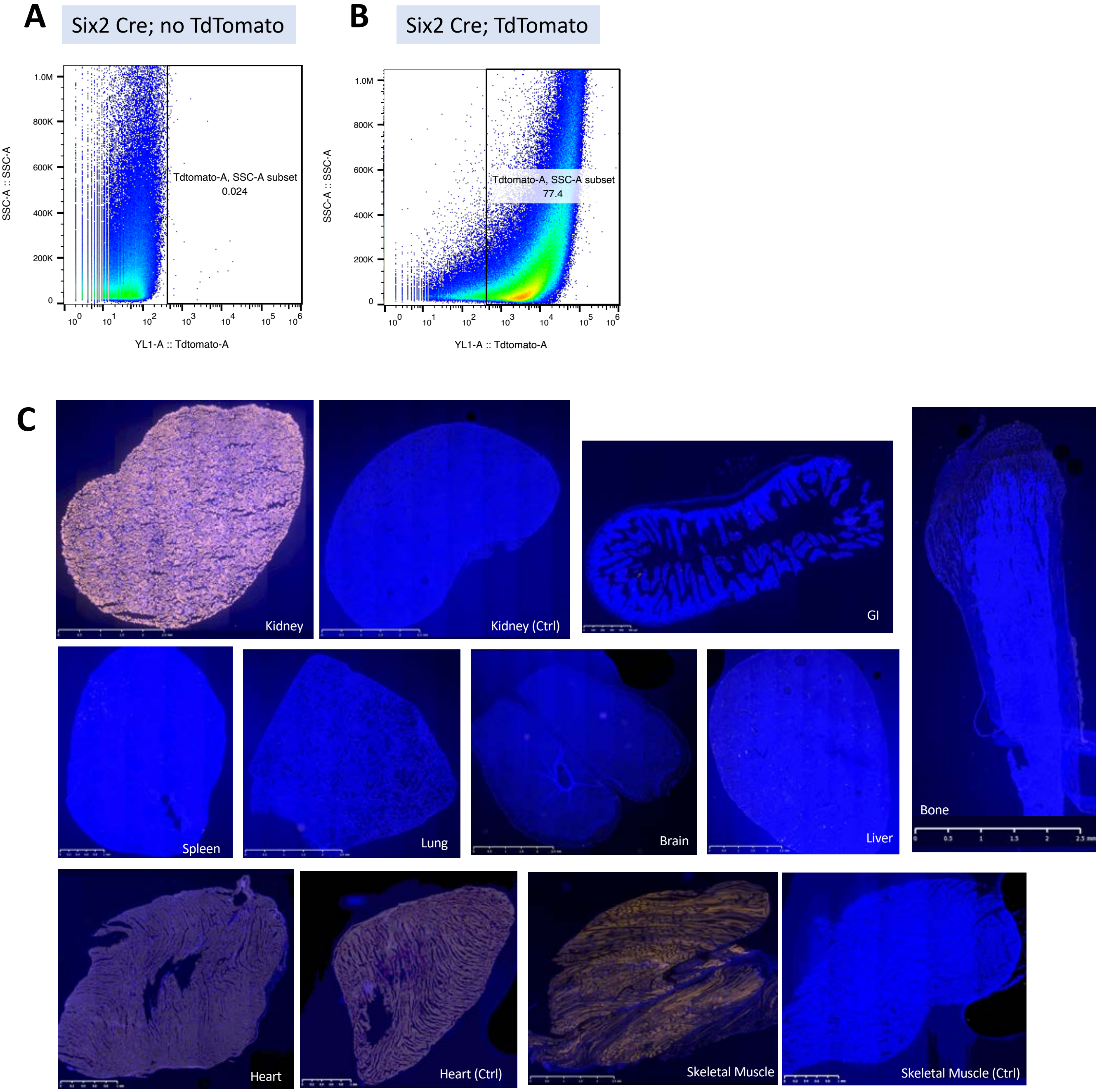

Supplemental Figure 4. Six2-Cre mice were crossed to Ai14 TdTomato reporter mice to visualize the Cre expression in kidney and its expression in different tissues in 11-week-old male mice. (A, B) Single cell suspensions from kidneys were generated for FACS analysis. TdTomato expression in various tissues is shown in (C). Scale bars represent 2.5 mm for all images, except that GI was 500μm, and 1mm for spleen, heart, and skeletal muscle (Ctrl).

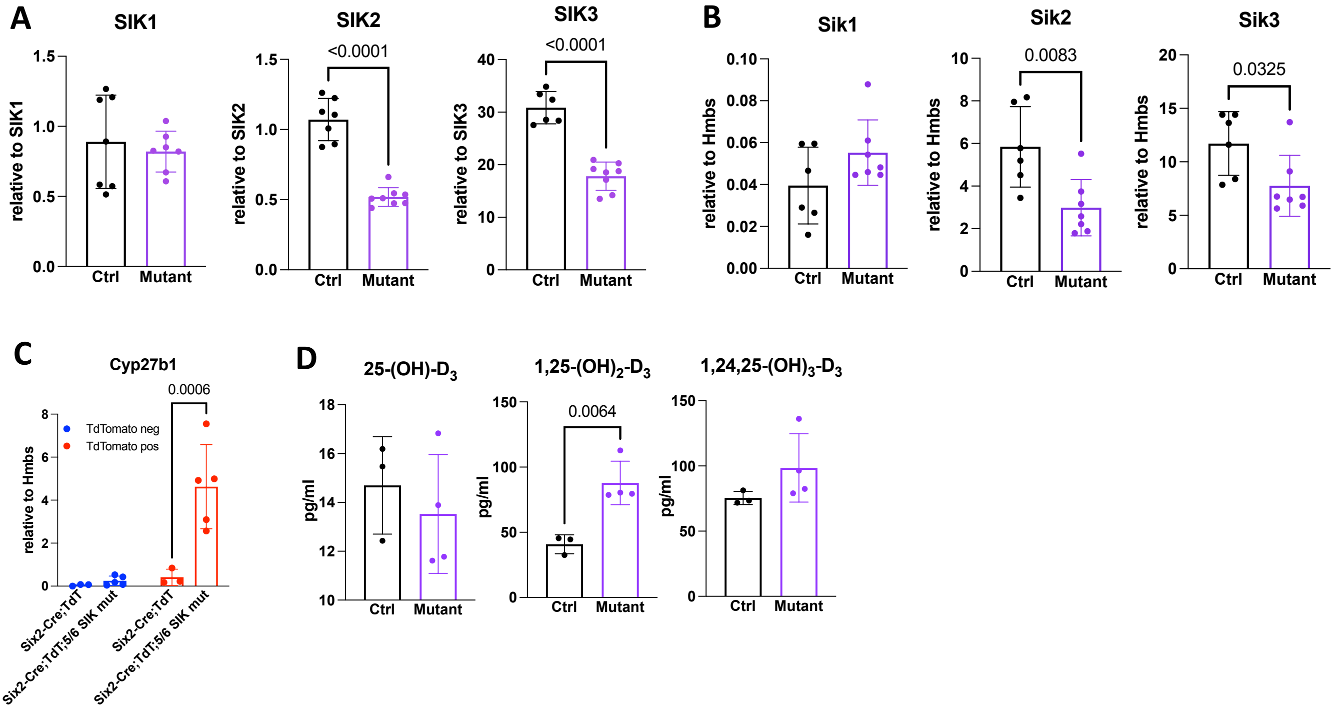

Supplemental Figure 5. (A) Whole kidney genomic DNA was isolated from control (*Sik1*$^{f/+}$;*Sik2*$^{f/f}$;*Sik3*$^{f/f}$) and 5 out of 6 SIK kidney-specific mutant mice (Six2-Cre; *Sik1*$^{f/+}$;*Sik2*$^{f/f}$;*Sik3*$^{f/f}$) and analyzed for SIK isoform deletion. (B) Whole kidney mRNA was isolated from control (*Sik1*$^{f/+}$;*Sik2*$^{f/f}$;*Sik3*$^{f/f}$) and 5 out of 6 SIK kidney-specific mutant mice (Six2-Cre; *Sik1*$^{f/+}$;*Sik2*$^{f/f}$;*Sik3*$^{f/f}$) and analyzed by RT-qPCR. (C) Increased *Cyp27b1* expression was confirmed in tdTomato positive cells isolated from kidney single cell suspensions of Six2-Cre; *Sik1*$^{f/+}$;*Sik2*$^{f/f}$;*Sik3*$^{f/f}$ mice crossed with Ai14 tdTomato reporter mice. (D) Vitamin D metabolites were measured by LC-MS/MS.

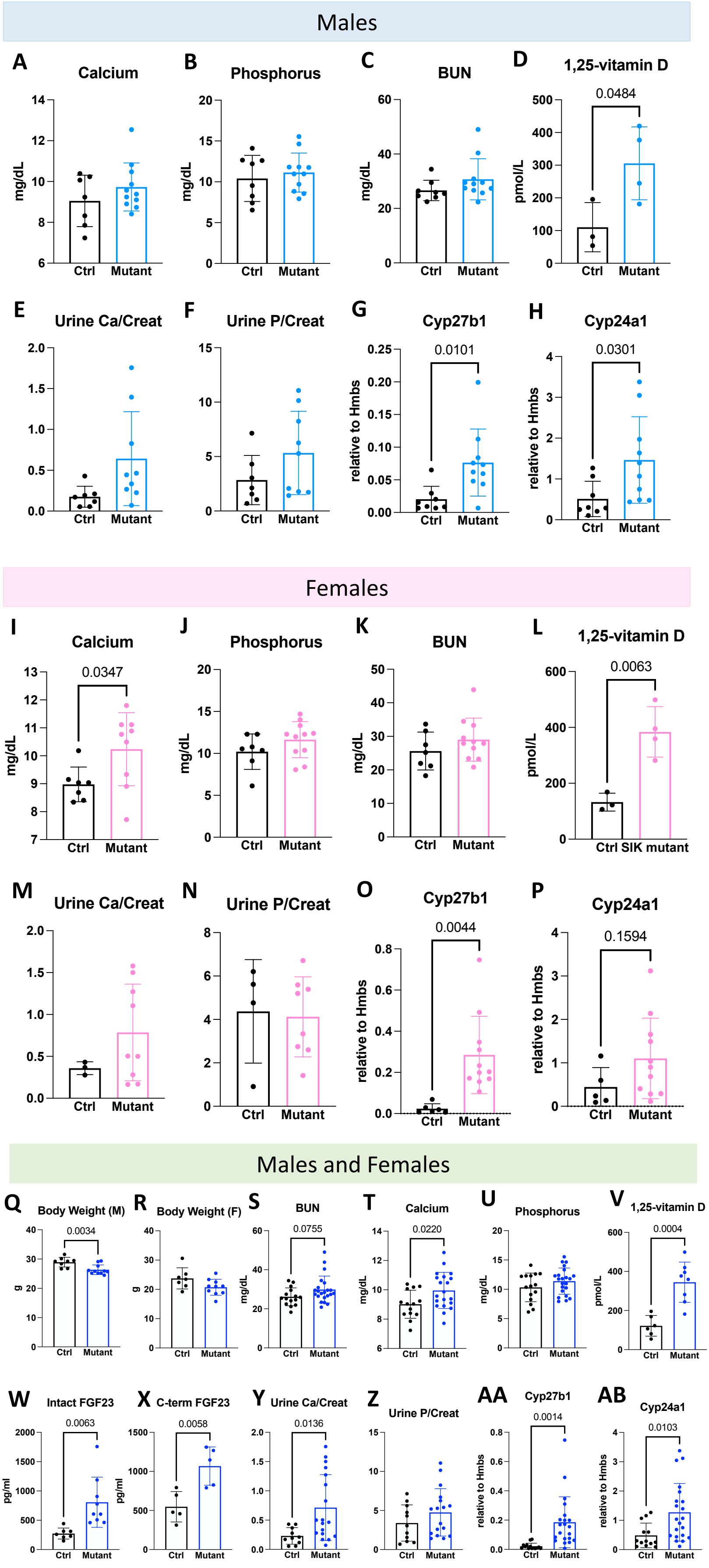

Supplemental Figure 6. Mineral metabolism phenotype in male (A-H) and female (I-P) in 3 month old Six2 Cre; *Sik1*$^{f/+}$;*Sik2*$^{f/f}$;*Sik3*$^{f/f}$ mice showed no sex effect. (Q-AB) The mineral metabolism phenotype in 3 month old Six2-Cre; *Sik1*$^{f/+}$;*Sik2*$^{f/f}$;*Sik3*$^{f/f}$ mice is comparable to what is seen in 6-8 week old animals.

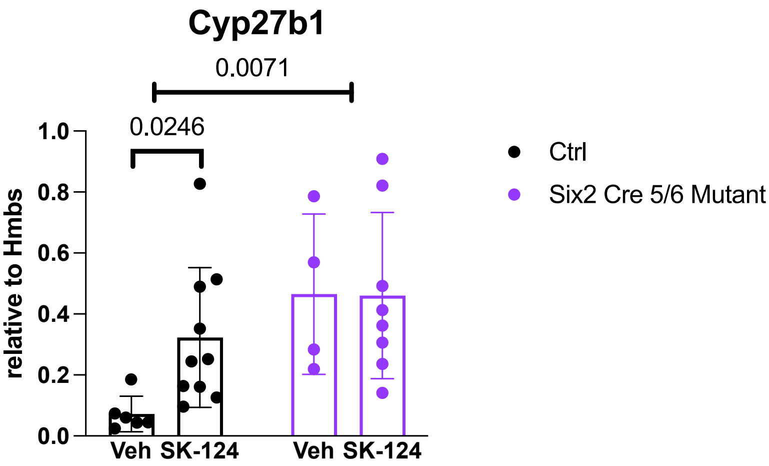

Supplemental Figure 7. *Cyp27b1* expression in kidneys from 6-8 week old Ctrl and Six2 Cre*; Sik1*$^{f/+}$;*Sik2*$^{f/f}$;*Sik3*$^{f/f}$ mice after acute treatment with vehicle or SK-124 (40 mg/kg; IP). Each dot represents each mouse and data are plotted as mean ± SD. Two-way ANOVA was performed followed by Sidak post-hoc test and p values are shown.

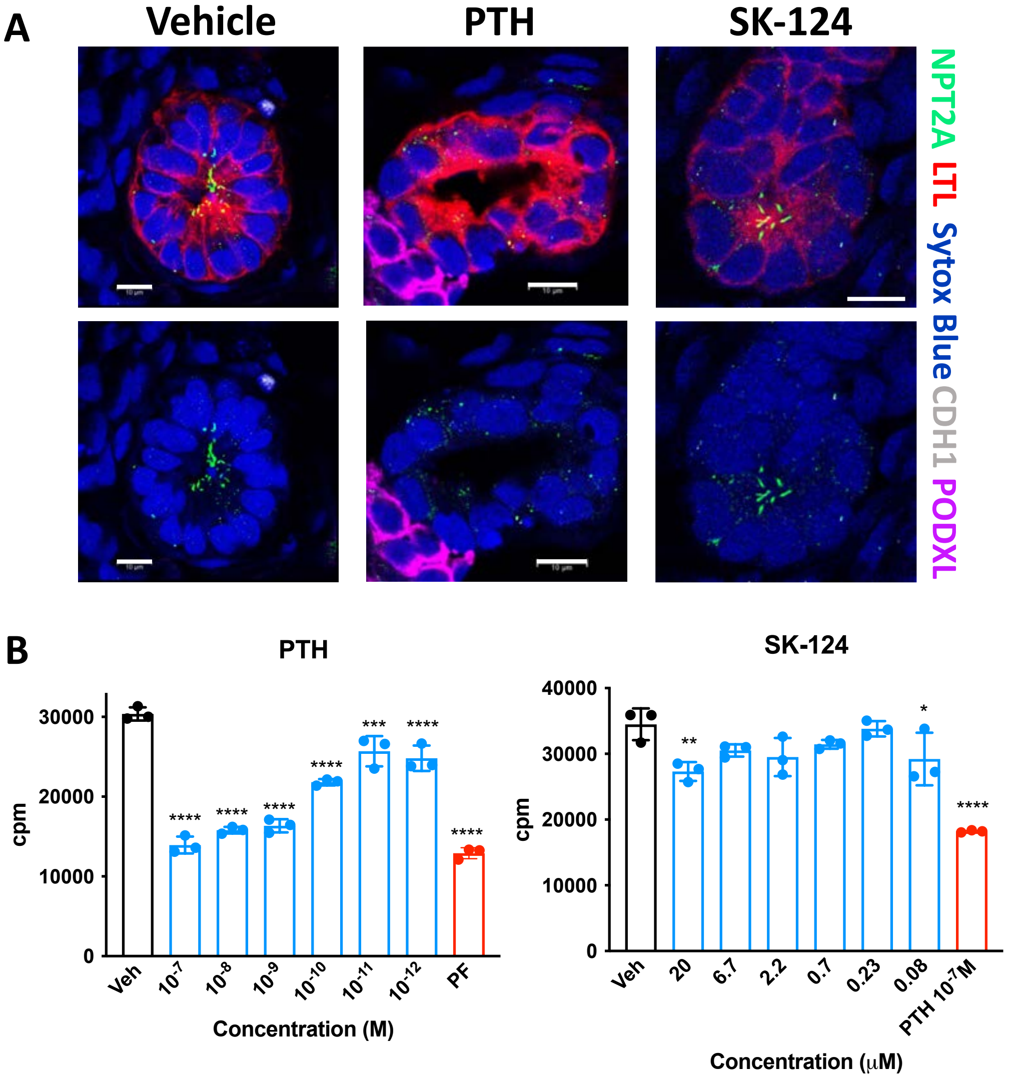

Supplemental Figure 8. (A) H9 human embryonic stem cell derived kidney organoids were immuno-stained with Npt2a antibody after treating with Veh, PTH (242 nM), or SK-124 (10 μM) for 30 min. White scale bars represent 10 μm (LTL (Lotus Tetragonolobus lectin) for proximal tubule; CDH1 for distal tubule; PODXL (Podocalyxin) for podocytes; Sytox Blue as the nuclear dye). (B) Phosphorus uptake assay was performed in opossum kidney (OK) cells using ^{32}P . A small molecule inhibitor for Npt2a, PF-06869206 (PF), was used as the positive control (10 μM). One-way ANOVA followed by Dunnett's post-hoc test against Veh was performed for statistical significance (* $p<0.05$; ** $p<0.01$, *** $p<0.001$, **** $p<0.0001$).

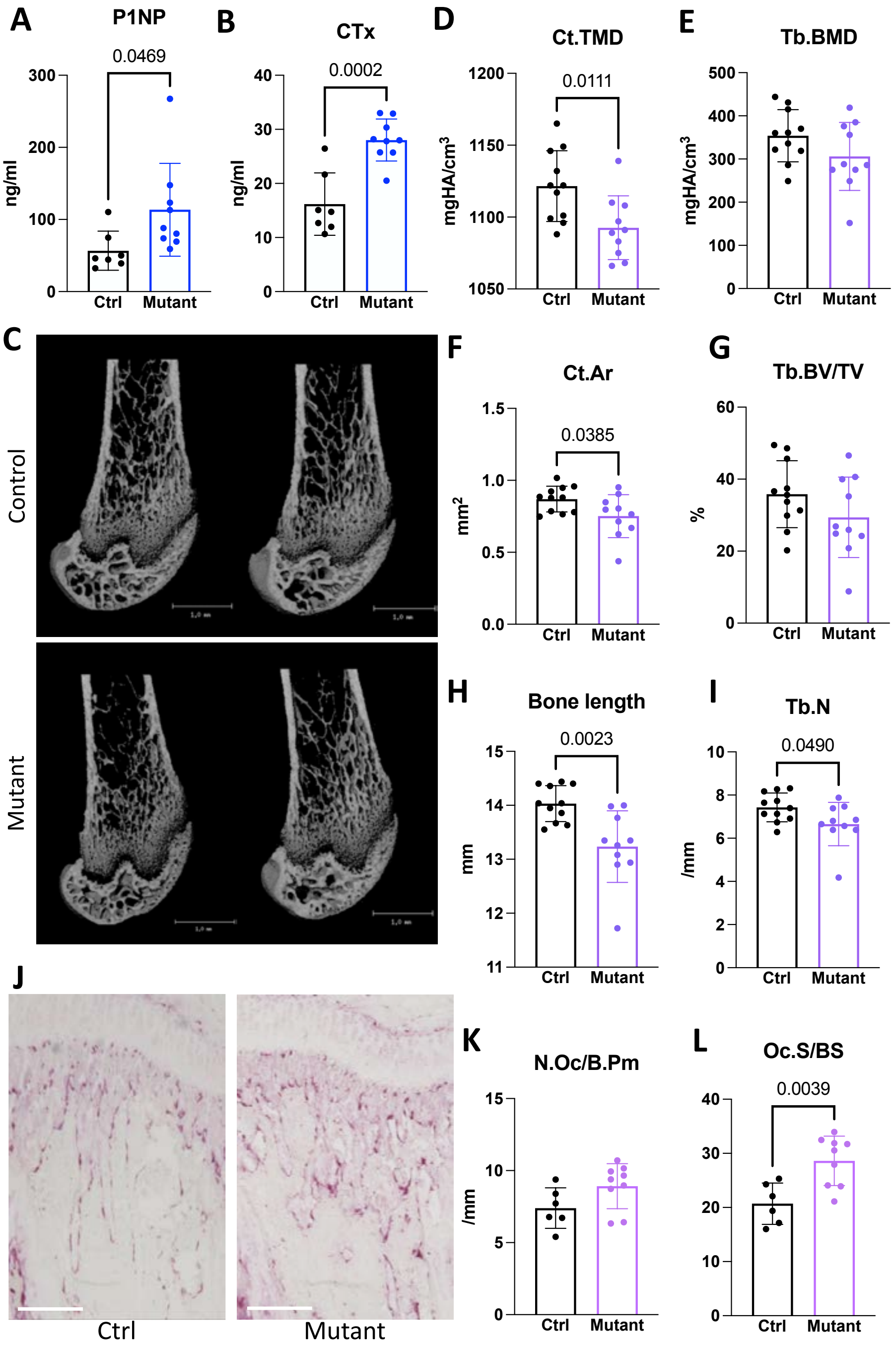

Supplemental Figure 9. Skeletal phenotypes were analyzed in 6 week-old male Six2 Cre; *Sik1*$^{f/+}$;*Sik2*$^{f/f}$;*Sik3*$^{f/f}$ mice and Cre-negative control littermates (*Sik1*$^{f/+}$;*Sik2*$^{f/f}$;*Sik3*$^{f/f}$). (A, B) Serum bone turnover markers P1NP (bone formation) and CTx (bone resorption). (C-I) MicroCT of the femur was formed. (J-L) TRAP (osteoclast) staining on paraffin-embedded tibia sections was performed. Representative photomicrographs are shown in (J) and the white scale bars represent 200 µm. Quantification of osteoclast numbers on trabecular bone surfaces is shown in (K, L). Student's t-tests were performed, all p values less than 0.05 are shown. See also Supplemental Table 9 for complete microCT data.

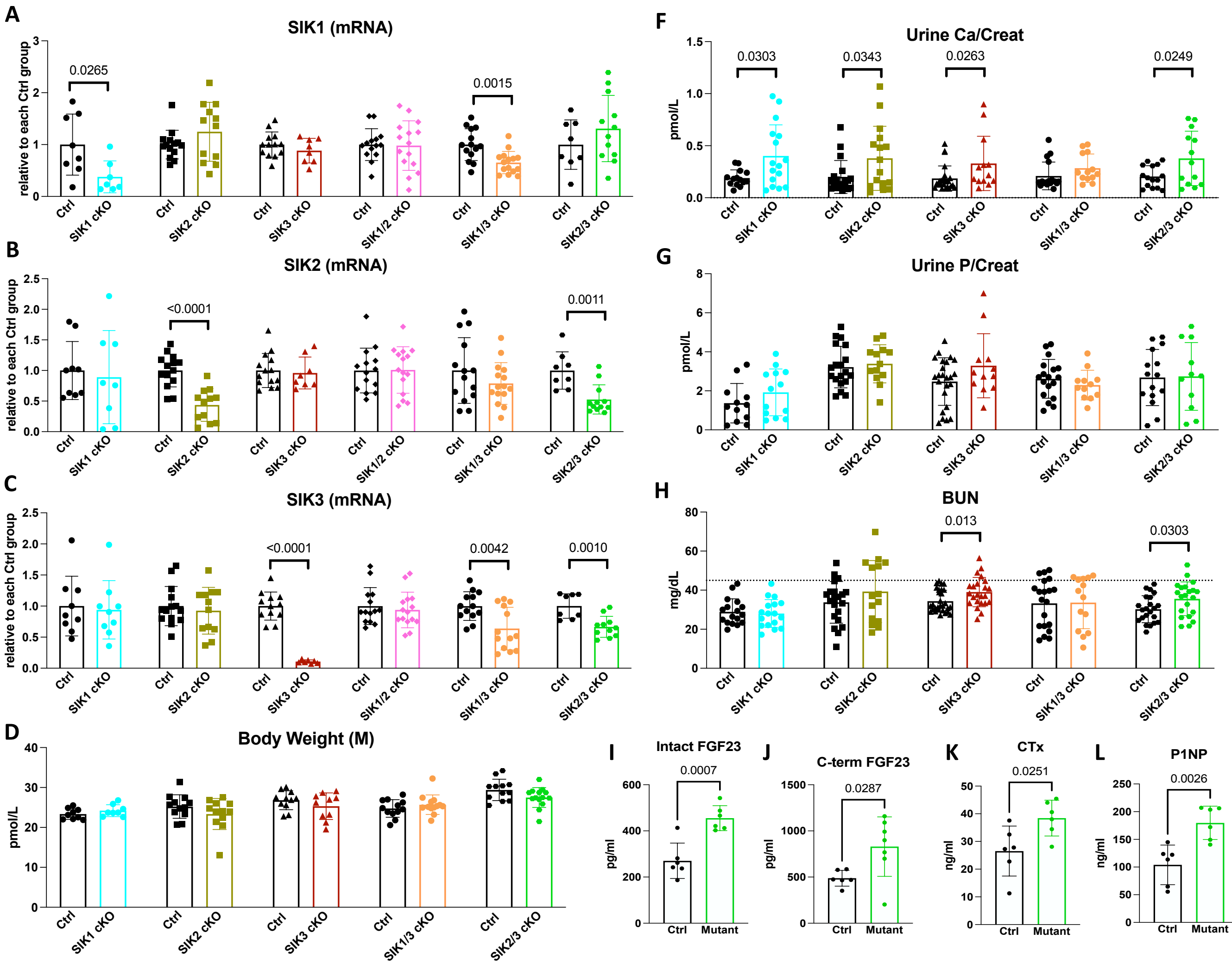

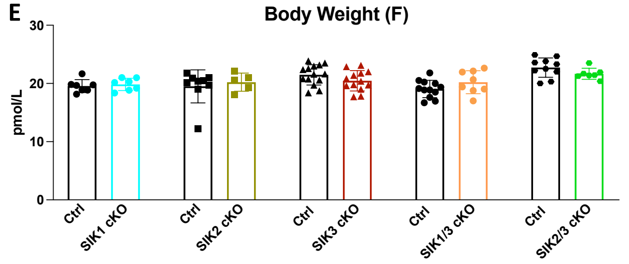

Supplemental Figure 10. (A-C) Whole kidney mRNA was isolated for RT-qPCR to confirm SIK gene deletion. (D, E) Body weights of 7-8 week-old male and female mice. (F, G) Urine calcium and phosphorus (normalized to urinary creatinine) of each strain is shown. (H) Serum BUN levels are shown, with a dotted line indicating the upper limit of the normal mouse BUN reference range. (I-L) Additional serum analysis of Six2-Cre; *Sik2*$^{f/f}$*;Sik3* $^{f/f}$ mice. T-tests were performed between control and mutant mice for each genotype, all p values less than 0.05 are shown.

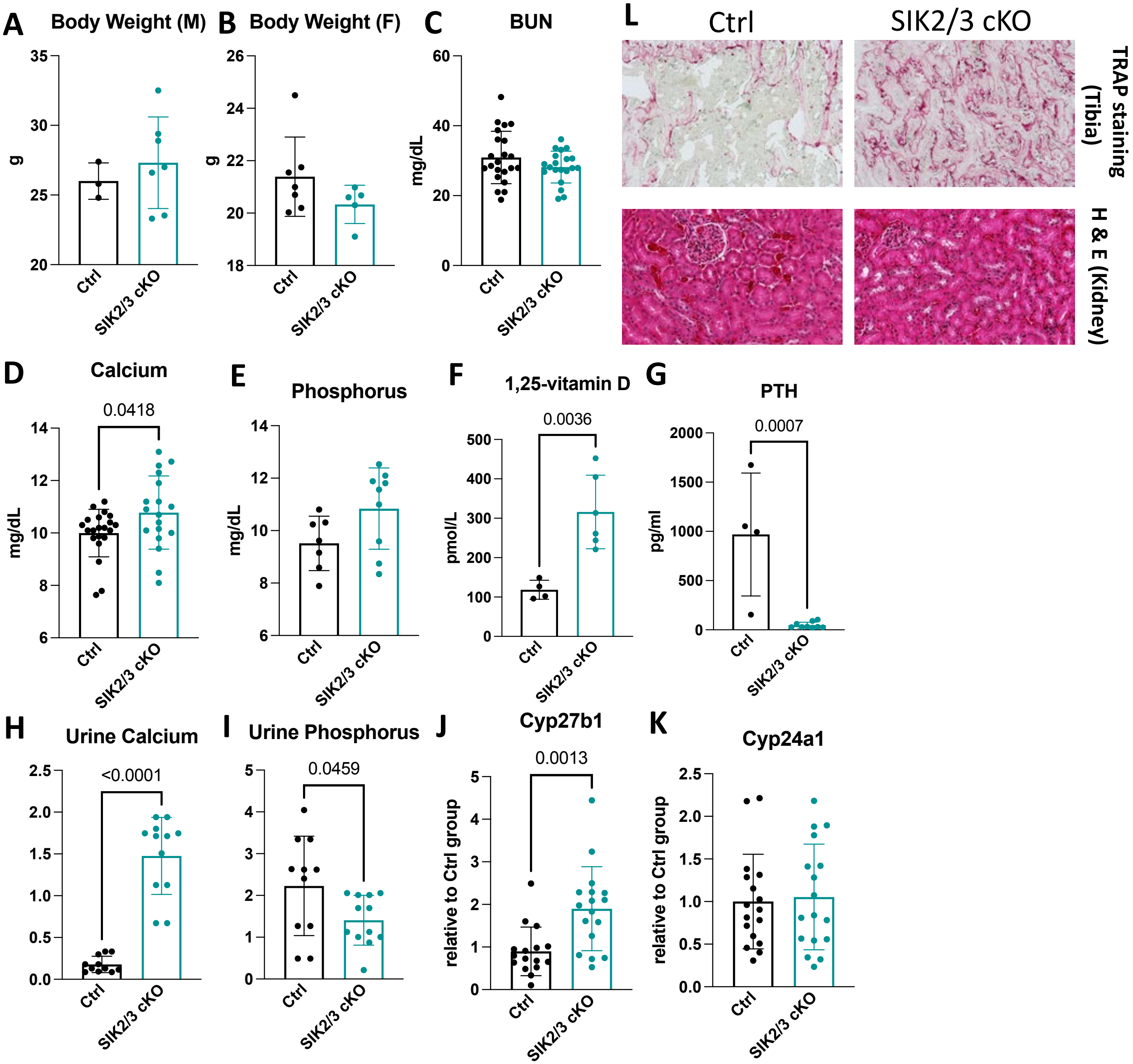

Supplemental Figure 11. 6 week old control (*Sik2*$^{f/f}$;*Sik3*$^{f/f}$) and SIK2/SIK3 cKO (Ubiquitin-Cre^{ERt2}; *Sik2*$^{f/f}$;*Sik3*$^{f/f}$) were treated with tamoxifen and then analyzed 2 weeks later. (A, B) Body weights at the time of sacrifice are shown for mice of both sexes. (C-G) Serum analyses were performed. (H, I) Urine calcium and phosphorus levels were measured and normalized to urine creatinine. (J, K) Kidney RNA was isolated for RT-qPCR. (L) Top: TRAP (red, osteoclast marker) staining in proximal tibia trabecular bone; bottom: kidney histopathology by H&E staining. Student's t tests were performed to compare control and mutant mice, with statistically-significant (p<0.05) differences shown on graphs.

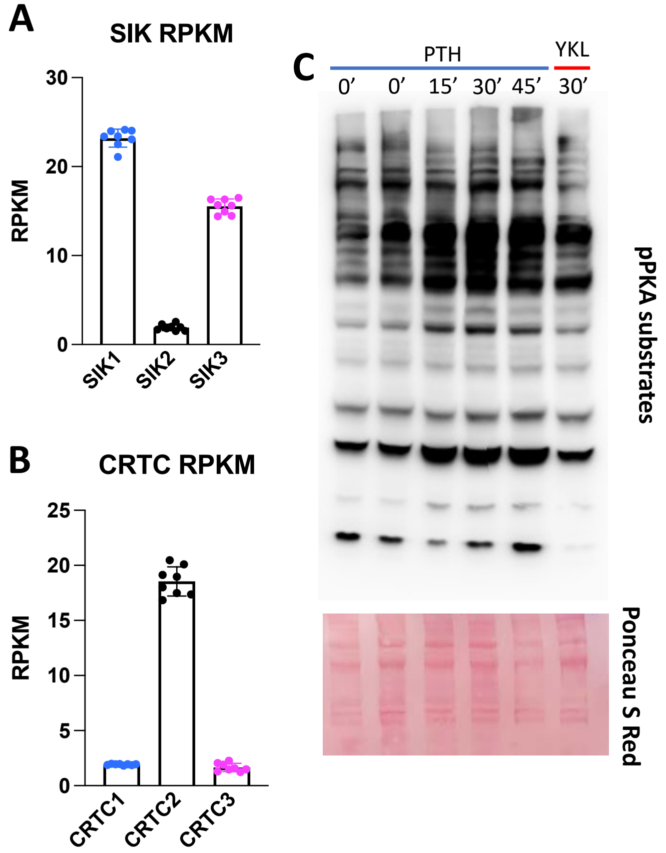

Supplemental Figure 12. (A, B) Whole kidney RNA-seq data was analyzed for SIK and CRTC isoform expression with RPKM units. (C) Immunoblotting with pPKA substrate antibody confirmed PTH responsiveness in opossum kidney (OK) cells. Ponceau S red stain is shown as loading control.

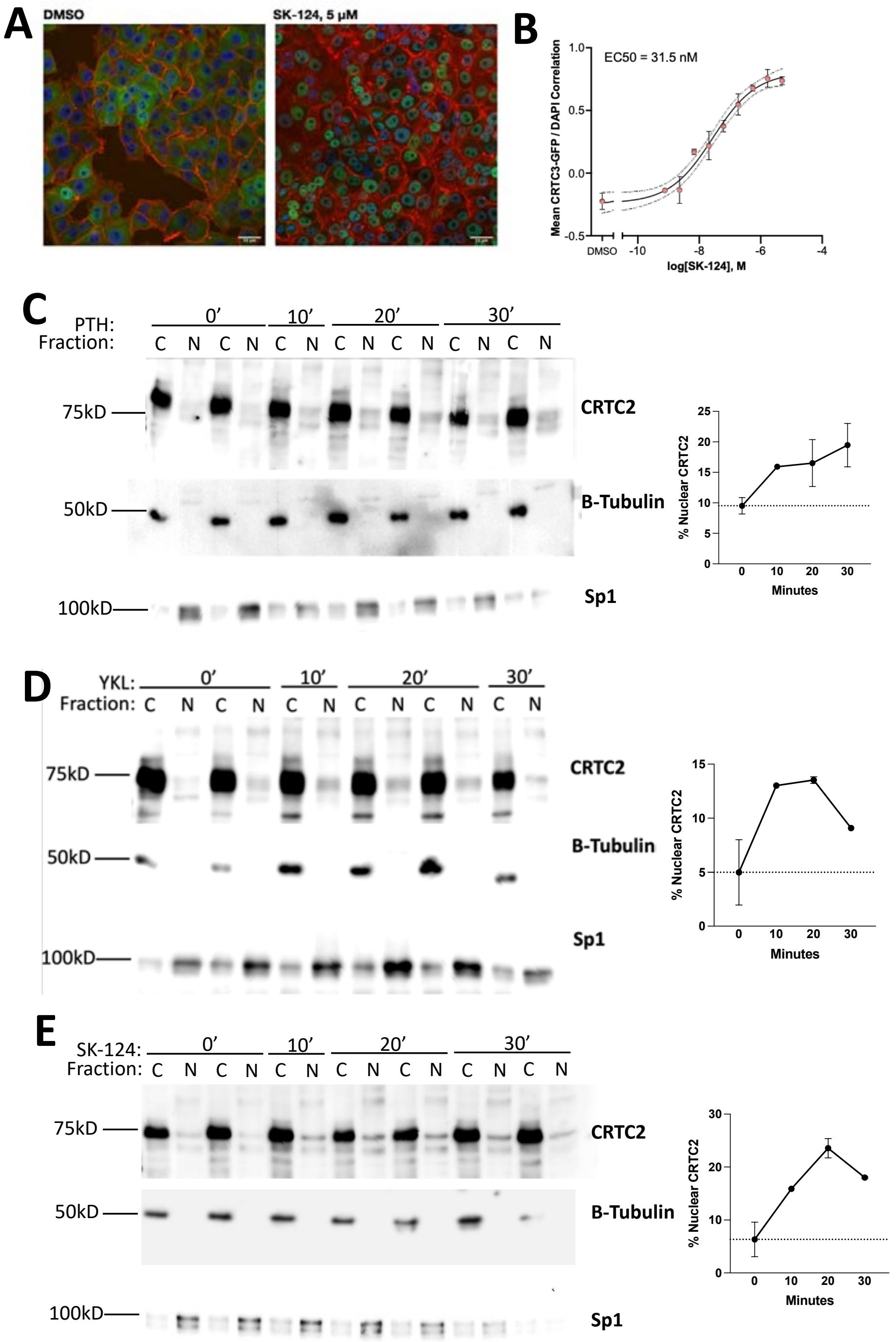

Supplemental Figure 13. (A, B) Doxycycline-inducible CRTC3-GFP containing Mel624 cells were treated with SK-124 (1 µM, 2 hr treatment) and assessed for CRTC3 nuclear translocation. Representative images are shown in A (white scale bars represent 33 µm) and the CRTC3-GFP nuclear translocation is shown as a dose-response curve in (B). Each data point represents an average correlation of DAPI and GFP signal in at least 100 cells. (C-E) OK cells were also treated for the indicated times with PTH , YKL-05-099 , and SK-124 followed by subcellular fractionation and immunoblotting. Replicates shown represent biological replicates. Graphs to the right of immunoblotting panels show quantification of CRTC2 nuclear fraction in each experiment.

A

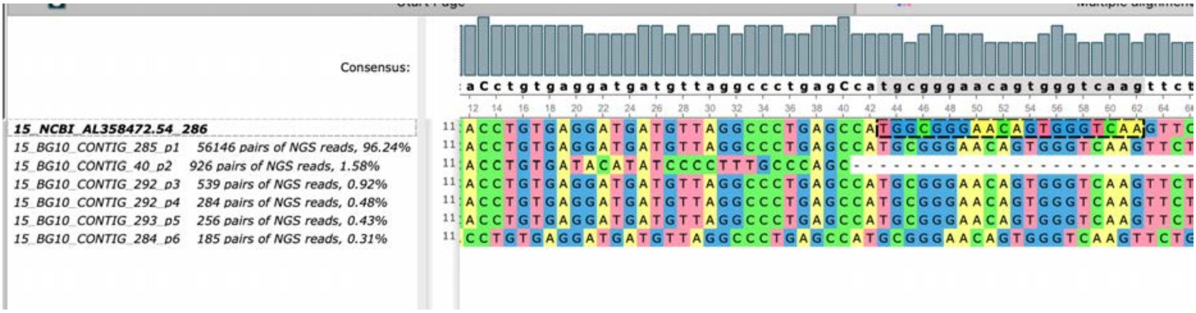

B

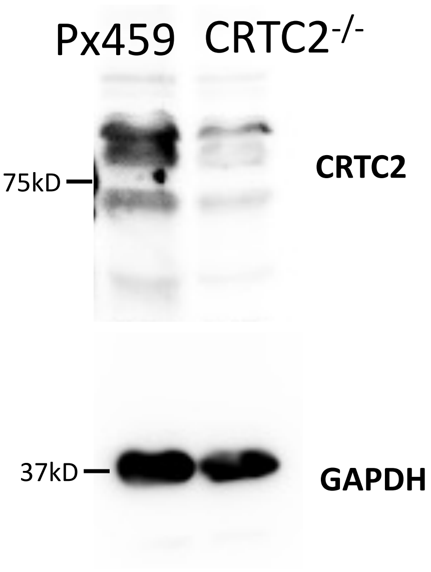

Supplemental Figure 14. (A) Screenshot of Unipro UGENE showing that one G is missing (at position 44) in monoclonal CRTC2 deficient H9 cells (2nd line) compared to original human CRTC2 sequence (1st line), resulting in homozygous, single nucleotide frame shift. (B) CRTC2 protein expression of monoclonal H9-CRTC2$^{-/-}$ cells compared to PX459 containing H9 cells. GAPDH blot is shown as the loading control.

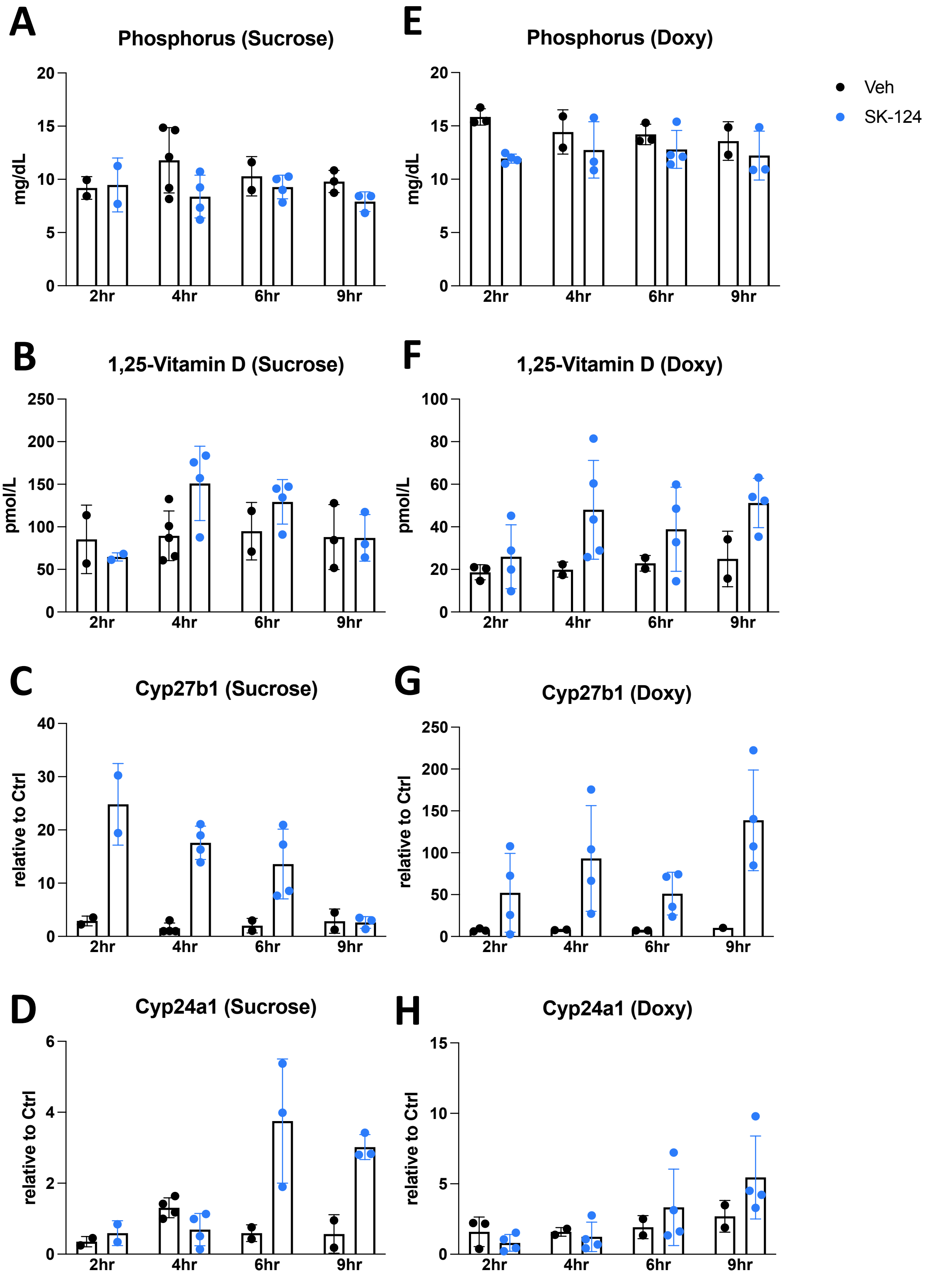

Supplemental Figure 15. iCTCF$^{pod-/-}$ mice were given sucrose or doxycycline-containing drinking water for 6.5 weeks. Then, animals were treated with either vehicle or SK-124 (40 mg/kg, IP) and euthanized 2, 4, 6, and 8 hours post injection, followed by serum collection (A, B, E, F) or kidney RNA isolation for RT-qPCR (C, D, G, H). Data are plotted as mean ± SD and each dot represents each mouse.

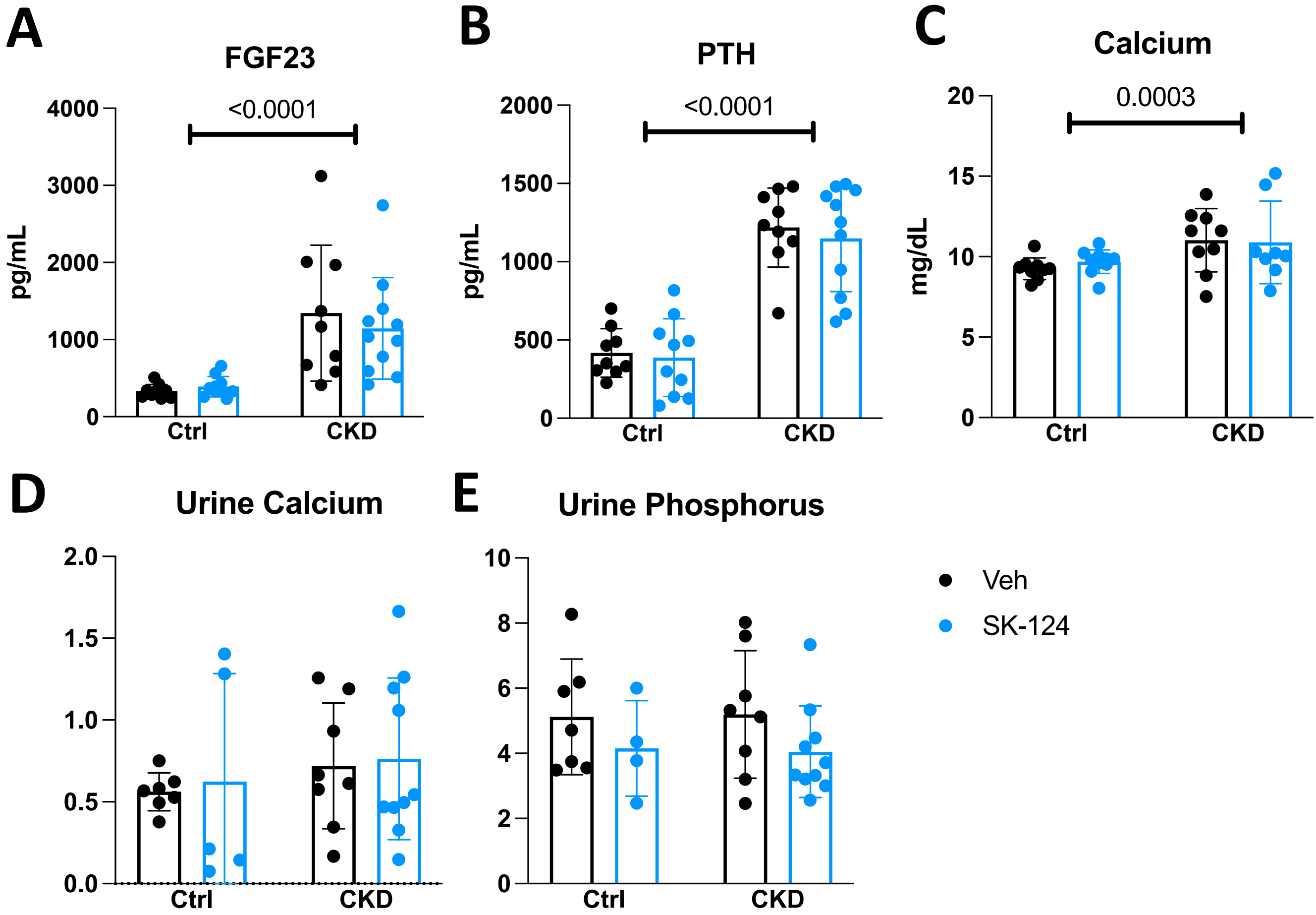

Supplemental Figure 16. Additional serum and urine parameters in 4-hour SK-124 treatment study in iCTCF$^{pod-/-}$ mice (Figure 8) are shown. (A, B) show increased FGF23 and PTH levels in CKD mice as expected, but not changed with SK-124 treatment. (C) Serum total calcium is shown. (D, E) Urine calcium and phosphorus normalized to creatinine are shown from urine samples collected at the time of sacrifice. Data are plotted as mean ± SD and each dot represents each mouse. 2-way ANOVA was performed to show significant CKD effects in A-C.

Full Unedited gel for Figure 7B

pCRTC2-S275

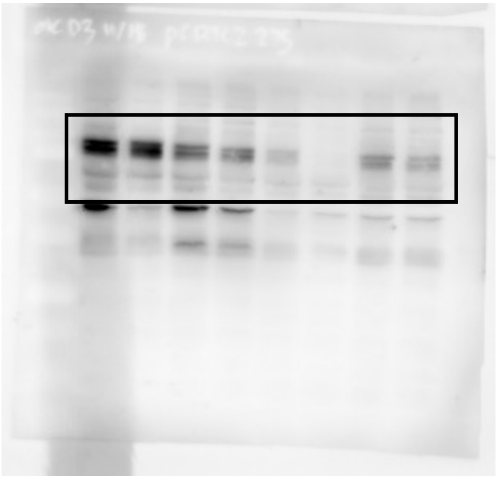

CRTC2

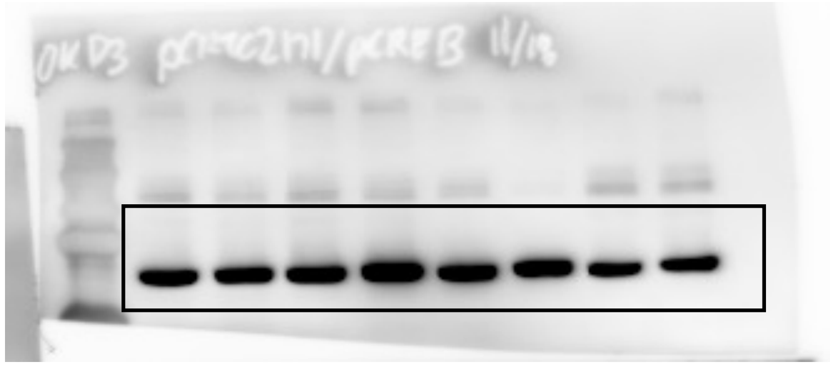

Full Unedited gel for Suppl Figure 12C bottom

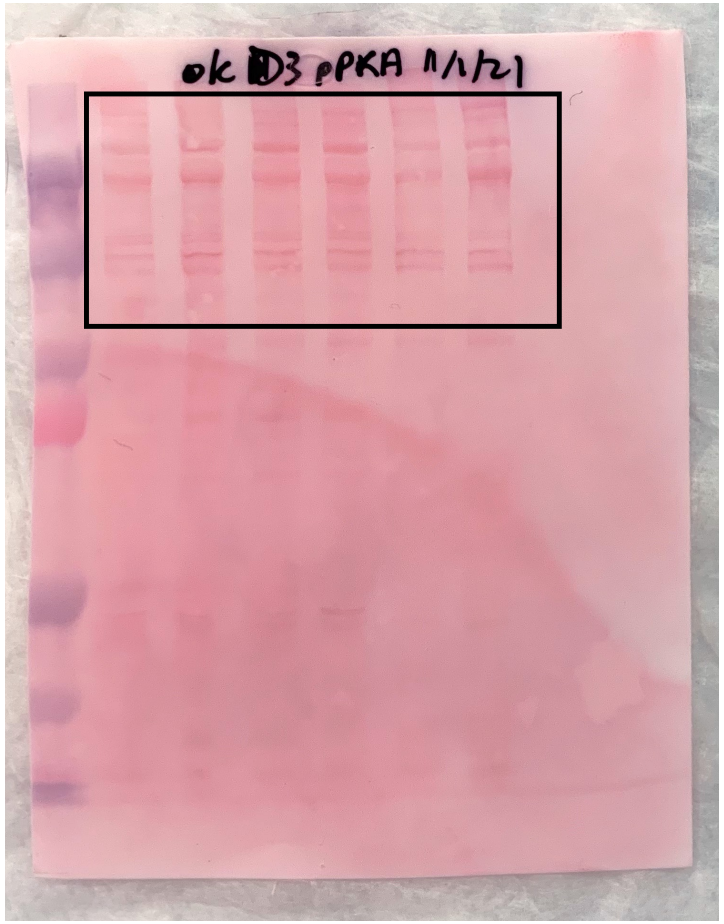

Ponceau S Red stain

Suppl Figure 12C top:
pPKA substrate blot presented in the figure is uncropped

Full Unedited gel for Supplemental Figure 13C

CRTC2

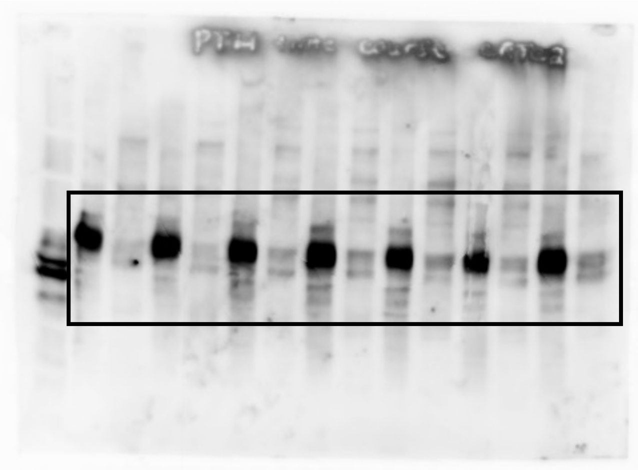

Sp-1

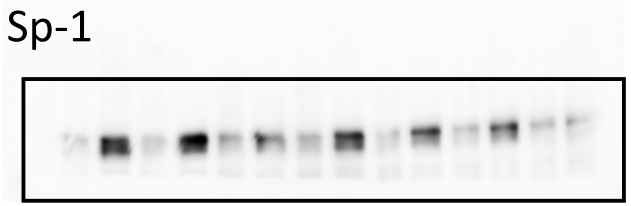

B-Tubulin

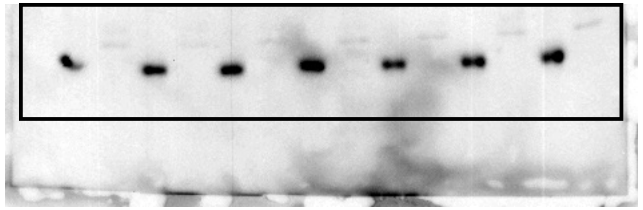

Full Unedited gel for Supplemental Figure 13D

CRTC2

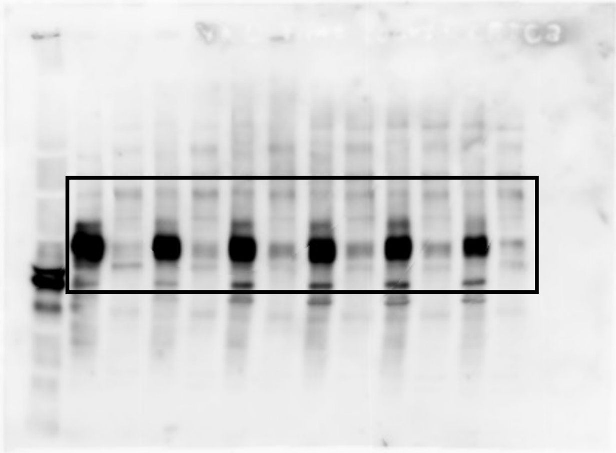

Sp-1

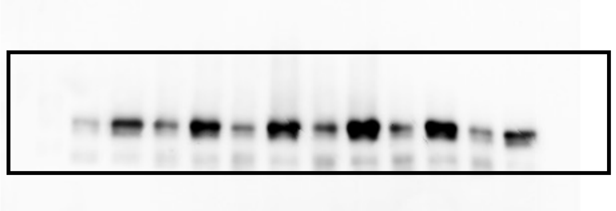

B-Tubulin

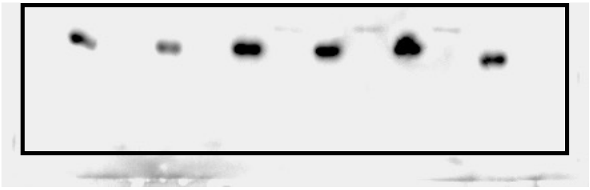

Full Unedited gel for Supplemental Figure 13E

CRTC2

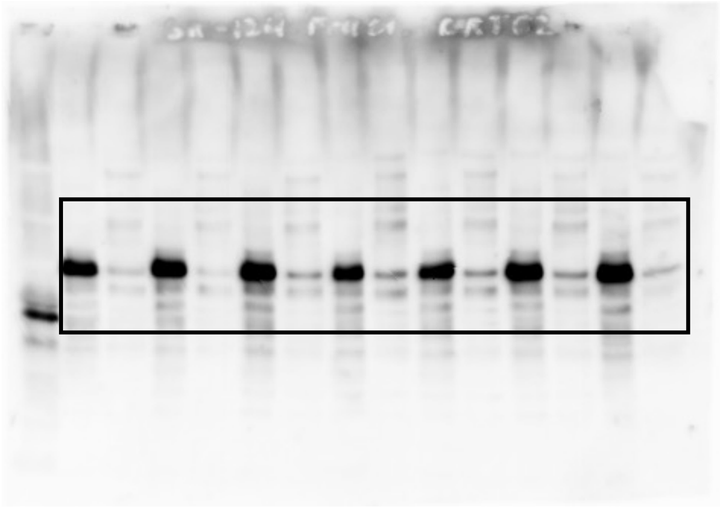

Sp-1

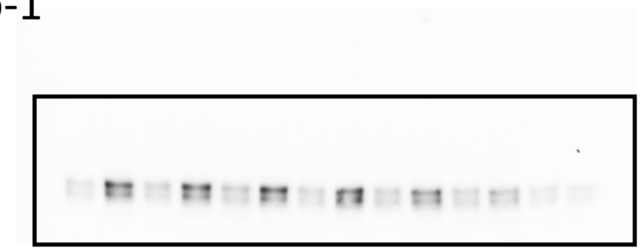

B-Tubulin

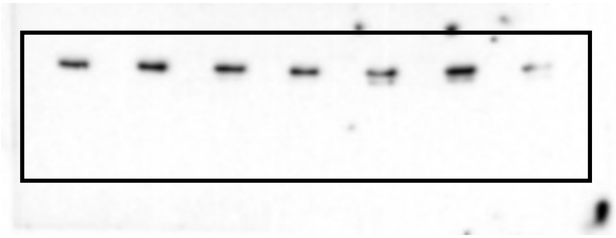

Full Unedited gel for Suppl Figure 14B

CRTC2

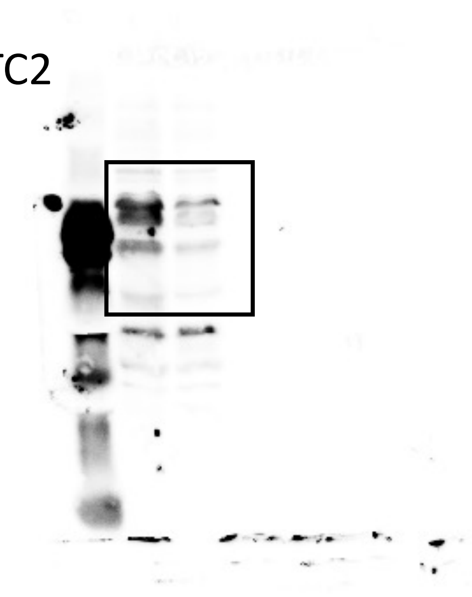

GAPDH

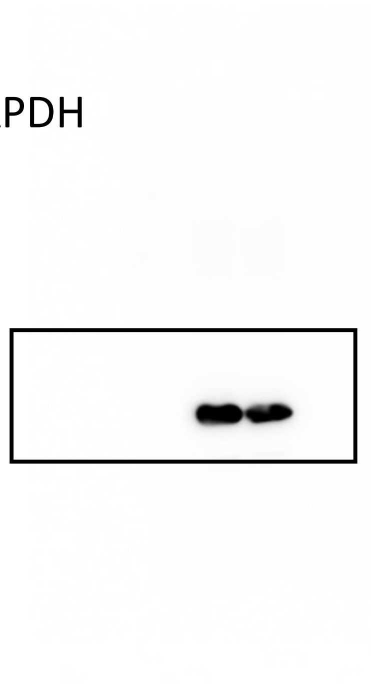

Supplement: Supplemental data [file jci-133-163627-s094.pdf]
